# Vitamin D-Related Gene Polymorphisms, Plasma 25-Hydroxy-Vitamin D, Cigarette Smoke and Non-Small Cell Lung Cancer (NSCLC) Risk

**DOI:** 10.3390/ijms17101597

**Published:** 2016-09-22

**Authors:** Xiayu Wu, Jiaoni Cheng, Kaiyun Yang

**Affiliations:** 1 School of Life Sciences, The Engineering Research Center of Sustainable Development and Utilization of Biomass Energy, Ministry of Education, Yunnan Normal University, Kunming 650500, China; 2 Yunnan Key laboratory for Basic Research on Bone and Joint Diseases, Yunnan Stem Cell Translational Research Center, Kunming University, Kunming 650500, China; jojo1988509@163.com; 3 Hospital of Kunming Medical College, Kunming 650101, China; yangky2214@163.com

**Keywords:** vitamin D, NSCLC, gene polymorphisms, risk, smoke

## Abstract

Epidemiological studies regarding the relationship between vitamin D, genetic polymorphisms in the vitamin D metabolism, cigarette smoke and non-small cell lung cancer (NSCLC) risk have not been investigated comprehensively. To search for additional evidence, the polymerase chain reaction-restriction fragment length polymorphism (PCR-RFLP) technique and radioimmunoassay method were utilized to evaluate 5 single-nucleotide polymorphisms (SNPs) in vitamin D receptor (*VDR*), 6 SNPs in 24-hydroxylase (*CYP24A1*), 2 SNPs in 1α-hydroxylase (*CYP27B1*) and 2 SNPs in vitamin D-binding protein (group-specific component, *GC*) and plasma vitamin D levels in 426 NSCLC cases and 445 controls from China. Exposure to cigarette smoke was ascertained through questionnaire information. Multivariable linear regressions and mixed effects models were used in statistical analysis. The results showed that Reference SNP rs6068816 in *CYP24A1*, rs1544410 and rs731236 in *VDR* and rs7041 in *GC* were statistically significant in relation to reduction in NSCLC risk (*p* < 0.001–0.05). No significant connection was seen between NSCLC risk and overall plasma 25-hydroxyvitamin D [25(OH)D] concentrations, regardless of smoking status. However, the mutation genotype of *CYP24A1* rs6068816 and *VDR* rs1544410 were also significantly associated with increased 25(OH)D levels only in both the smoker and non-smoker cases (*p* < 0.01–0.05). Meanwhile, smokers and non-smokers with mutated homozygous rs2181874 in *CYP24A1* had significantly increased NSCLC risk (odds ratio (OR) = 2.14, 95% confidence interval (CI) 1.47–3.43; *p* = 0.031; OR = 3.57, 95% CI 2.66–4.74; *p* = 0.019, respectively). Smokers with mutated homozygous rs10735810 in *VDR* had significantly increased NSCLC risk (OR = 1.93, 95% CI 1.41–2.76; *p* = 0.015). However, smokers with mutated homozygous rs6068816 in *CYP24A1* had significantly decreased NSCLC risk (OR = 0.43, 95% CI 0.27–1.02; *p* = 0.006); and smokers and non-smokers with mutated homozygous rs1544410 in *VDR* had significantly decreased NSCLC risk (OR = 0.51, 95% CI 0.34–1.17; *p* = 0.002; OR = 0.26, 95% CI 0.20–0.69; *p* = 0.001, respectively). There are significant joint effects between smoking and *CYP24A1* rs2181874, *CYP24A1* rs6068816, *VDR* rs10735810, and *VDR* rs1544410 (*p* < 0.01–0.05). Smokers with mutated homozygous rs10735810 in *VDR* had significantly increased NSCLC risk (OR = 1.93, 95% CI 1.41–2.76; *p* = 0.015). In summary, the results suggested that the lower the distribution of vitamin D concentration, the more the genetic variations in *CYP24A1*, *VDR* and *GC* genes may be associated with NSCLC risk. In addition, there are significant joint associations of cigarette smoking and vitamin D deficiency on NSCLC risk.

## 1. Introduction

**Figure 1:**
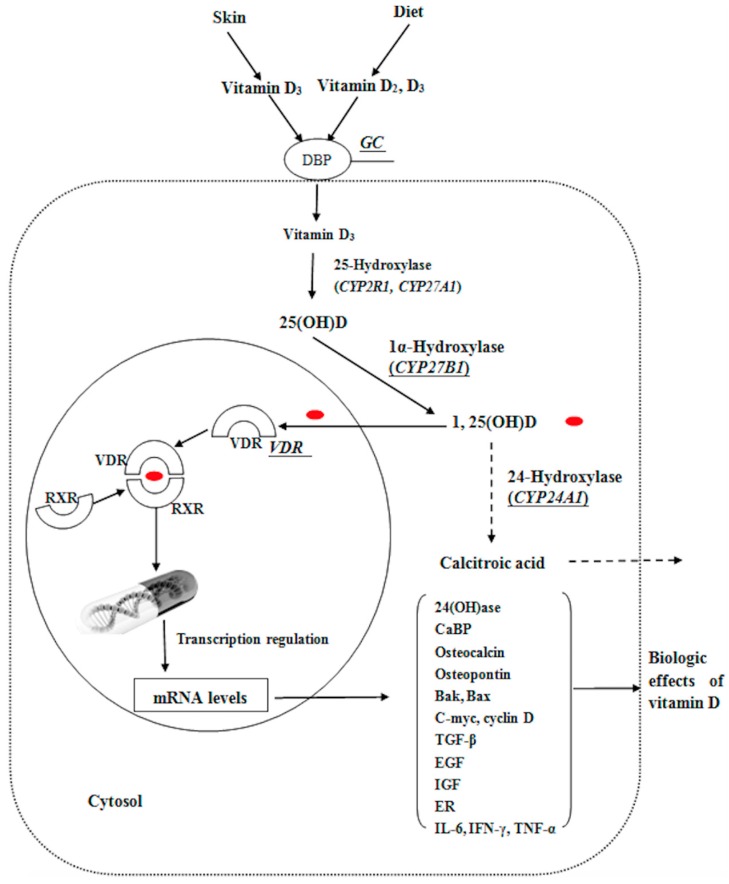
Physiological roles of vitamin D-binding protein (encoded by the *group-specific component*, *GC* gene), 1-hydroxylase (*CYP27B1*), 24-hydroxylase (*CYP24A1*), and vitamin D receptor (*VDR*) genes in vitamin D mechanism. Vitamin D–binding protein (DBP); Retinoid X receptor (RXR); 24-Hydroxylase [24(OH)ase]; Ca^2+^-buffer protein (CaBP); Proapoptotic B cell chronic lymphocytic leukemia/lymphoma (BCL-2); BCL-2 antagonist killer (Bak); BCL-2-associated X (Bax); Transforming growth factor-β (TGF-β); Epidermal growth factor (EGF); Insulin-like growth factor (IGF); Endoplasmic reticulum (ER); Interleukin-6 (IL-6); Interferon-γ (IFN-γ); Tumor necrosis factor-α (TNF-α). Red dots represent 1,25(OH)D.

Non-small cell lung cancer (NSCLC), one of the most common and highly frequent malignancies, is a leading cause of cancer-related death in the population [1]. Risk factors associated with environment and lifestyle include family history of lung cancer, history of pulmonary diseases, nutrition, air pollution, cigarette smoke, and exposure to radiation, asbestos and radon. Furthermore, recent epidemiological studies have shown that exposure to solar radiation (in particular ultraviolet B (UVB) radiation) and vitamin D intake is connected with decreased incidence of many cancers including lung, breast, prostate and colon cancer [2,3,4,5,6]. The mortality of lung cancer is lowest during the autumn and summer months, which are associated with the highest vitamin D levels in a year [7]. The vitamin D_3_ and vitamin D_2_, two natural primary forms of vitamin D, are endogenously generated from sun exposure or ingestion of food or supplements [8]. Vitamin D is hydroxylated at 25 position to 25-hydroxy vitamin D [25(OH)D] and further hydroxylated by 1α-hydroxylase (encoded by *CYP27B1*) in the kidney to 1,25-dihydroxy vitamin D [1,25(OH)2D]. The concentrations of 25(OH)D are usually 100 to 1000-fold higher than the ones of 1,25(OH)_2_D [9]. It is released into the blood circulation in the gut and then passively reabsorbed in the kidneys through mechanisms mediated by the vitamin D receptor (encoded by *VDR* gene), as shown in Figure 1.

Finally, 24-hydroxylase (encoded by *CYP24A1*) degrades both 1,25(OH)_2_D and 25(OH)D into non-active forms. *VDR*, the superfamily of transacting transcriptional regulatory factors, adjusts to several gene transcriptions, including cell apoptosis, pro-oncogenes, immunomodulation, differentiation, and tumor-suppressor genes [9,10]. The vitamin D-binding protein (encoded by the *group-specific component*, *GC* gene) mainly promotes transportation of vitamin D metabolites [11]. Laboratory studies showed that high 1,25(OH)_2_D levels can inhibit differentiation and proliferation in human lung cancer cell lines [12], and circulating 25(OH)D level may predict early-stage NSCLC patients’ survival [13,14]. Vitamin D-related genes have highly polymorphic genotypes in different human populations. Moreover, as a subgroup, they have been widely analyzed in multitudinous cancer-related studies [15,16]. However, in previously conducted studies, genetic variation in *VDR* has not been systematically analyzed with regard to NSCLC, and very limited data are available on *CYP27B1* and *CYP24A1* polymorphisms [17,18]. In addition, epidemiological and clinical studies inspecting the associations between NSCLC risk and vitamin D status are limited in number and inconclusive [19,20]. To investigate the associations between vitamin D, genetic polymorphisms in the vitamin D metabolism pathway, cigarette smoke and NSCLC risk, we conducted a case-control study and utilized the polymerase chain reaction-restriction fragment length polymorphism (PCR-RFLP) technique to evaluate the single-nucleotide polymorphisms (SNPs), which were located in the protein coding and promoter regions *VDR*, *CYP24A1*, *CYP27B1* and *GC* genes. We then evaluated whether the vitamin D status was connected with the NSCLC risk by a radioimmunoassay method. We additionally examined the deficiency of vitamin D combined with smoking through questionnaire information.

## 2. Results

### 2.1. Descriptive Characteristics

Characteristics of controls and NSCLC cases are displayed in Table 1. There were no statistically significant differences in the allocation of age, gender, marriage status, body mass index (BMI), education, and leisure physical activity between cases and controls. However, the significant differences were observed in smoking status and family history of cancer between cases and controls (17.8% vs. 9.5%, *p* < 0.001). As expected, more NSCLC cases were smokers compared to the controls (70.0% vs. 48.5%, *p* < 0.001).

### 2.2. Relationship between SNPs, Smoking and NSCLC Risk

Fifteen SNPs in four genes were examined in our study. The odds ratios (ORs) and 95% confidence intervals (CIs) for association between smoking and NSCLC were stratified by 25(OH)D, as shown in Table 2. The results showed that smoking was related to increased NSCLC risk, regardless of the concentration of plasma 25(OH)D <20 ng/mL or ≥20 ng/mL (OR = 2.74, 95% CI 1.97–3.01, *p* < 0.001 in <20 ng/mL; OR = 2.48, 95% CI 2.05–2.97, *p* < 0.001 in ≥20 ng/mL, respectively), in a statistically significant manner. The OR and 95% CIs for associations between vitamin D-related genotypes and NSCLC risk are shown in Table 3. For *CYP24A1* polymorphisms, we found that rs6068816 was significant related to reduction of NSCLC risk (TT vs. CC, OR = 0.31, 95% CI 0.21–0.47; *p* < 0.001). No statistically significant increased risk of NSCLC was observed in rs2181874 (AA vs. GG, OR = 1.40, 95% CI 0.85–1.92, *p* = 0.07) and rs2296241 (AA vs. GG, OR = 1.27, 95% CI 0.76–1.55, *p* = 0.09), respectively. For *VDR* polymorphisms, *bovine submaxillary mucin* (*Bsm1*) (rs1544410) and *Taq1* (rs731236), were associated with reduction in risk of NSCLC, (*AA* vs. *GG*, OR = 0.71 95% CI 0.68–0.96; *p* = 0.032; CC vs. TT, OR = 0.84, 95% CI 0.56–0.98, *p* = 0.037, respectively). For *GC* gene polymorphisms, we also found that there was a statistically significant reduction of NSCLC risk in rs7041 (TT vs. GG, OR = 0.61, 95% CI 0.41–0.93; *p* < 0.001). Moreover, we did not observe any significant impact of *CYP27B1* polymorphisms on risk of NSCLC. After we adjusted for multiple comparisons, none of the SNP-NSCLC risk *p* values were <0.002 and the threshold was determined using the Bonferroni correction.

### 2.3. Plasma 25(OH)D Concentrations with Different Genotypes

The overall plasma 25(OH)D concentrations were slightly higher in the controls than in the other cases, but no statistically significant difference between them was observed (Table 1). When correlations between SNPs and plasma 25(OH)D concentrations in cases and controls were tested, the *CYP24A1* SNP rs6068816 and *VDR* SNP rs1544410 were significantly associated with increased 25(OH)D concentration. For CC, CT, TT in rs6068816 and GG, GA, AA in rs1544410 genotypes, the means and standard deviations (SD) of plasma 25(OH)D were 16.4 ± 4.5, 20.7 ± 7.3, 25.7 ± 10.3 ng/mL (*p* = 0.007) and 16.5 ± 8.6, 20.7 ± 6.8, 25.2 ± 7.3 ng/mL (*p* = 0.009), respectively. However, the differences were not significant in the controls (Table 4).

### 2.4. Joint Association between Vitamin D-Related Polymorphisms and Plasma 25(OH)D on NSCLC Risk

We noted an effect modification on NSCLC risk (*p* ≤ 0.01–0.05) for *CYP24A1* rs6068816, *VDR* rs10735810 and *VDR* rs1544410 polymorphisms. Homozygous for the common allele of *CYP24A1* rs6068816, *VDR* rs10735810, and *VDR* rs1544410 in patient women, with their plasma 25(OH)D of ≥20 ng/mL, had a reduced NSCLC risk compared to patient women with plasma 25(OH)D <20 ng/mL (OR = 0.48, 95% CI 0.25–0.65; OR = 0.46, 95% CI 0.31–0.63; OR = 0.52, 95% CI 0.29–0.97, respectively, in Table 5). With adjustment for multiple comparisons, none of the interaction *p* values were below the Bonferroni-determined threshold.

### 2.5. Plasma 25(OH)D Concentrations in Smokers and Non-Smokers with Different Genotypes

In the subgroup, when we compared plasma 25(OH)D concentrations with genotypes in smokers and non-smokers for both cases and control groups, the mutation genotype of *CYP24A1* rs6068816 and *VDR* rs1544410 were also significantly associated with increased 25(OH)D levels in both smokers and non-smokers in the cases group. For CC, CT, TT in rs6068816 and GG, GA, AA in rs1544410 genotypes, the means and standard deviations of plasma 25(OH)D were 14.7 ± 5.2, 16.3 ± 7.8, 18.7 ± 5.8 ng/mL (*p* = 0.02) in smokers with rs6068816, 18.4 ± 4.7, 22.4 ± 6.9, 27.2 ± 13.4 ng/mL (*p* = 0.003) in non-smokers with rs6068816, and 14.5 ± 7.8, 18.5 ± 9.6, 25.6 ± 7.0 ng/mL (*p* = 0.003) in smokers with rs1544410, 18.9 ± 10.7, 21.6 ± 4.7, 25.1 ± 7.9 (*p* = 0.01) in non-smokers with rs1544410, respectively, in the cases group. However, the differences were also not significant in smokers and non-smokers of the control group (Table 6).

### 2.6. Interaction between Gene and Smoking

When the stratified data in subgroups of subjects by cigarette smoke status were analyzed, we found that smokers and non-smokers with mutated homozygous rs2181874 in *CYP24A1* had significantly increased NSCLC risk (OR = 2.14, 95% CI 1.47–3.43; *p* = 0.031; OR = 3.57, 95% CI 2.66–4.74; *p* = 0.019, respectively). However, smokers with mutated homozygous rs6068816 in *CYP24A1* had significantly decreased NSCLC risk (OR = 0.43, 95% CI 0.27–1.02; *p* = 0.006). Smokers with mutated homozygous rs10735810 in *VDR* had significantly increased NSCLC risk (OR = 1.93, 95% CI 1.41–2.76; *p* = 0.015); smokers and non-smokers with mutated homozygous rs1544410 in *VDR* had significantly decreased NSCLC risk (OR = 0.51, 95% CI 0.34–1.17; *p* = 0.002; OR = 0.26, 95% CI 0.20–0.69; *p* = 0.001, respectively). Logistic regression analyses showed significant joint effects between smoking and *CYP24A1* rs2181874 (*p* = 0.016), *CYP24A1* rs6068816 (*p* = 0.036), *VDR* rs10735810 (*p* = 0.004), and *VDR* rs1544410 (*p* = 0.002). Detailed data are shown in Table 7.

## 3. Discussion

The findings from our study did not provide evidence that higher 25(OH)D levels are related to declined NSCLC risk. These results differ from those of the Finnish study, which found that there were no associations in men or older people, while there was an opposite association between lung cancer risk and 25(OH)D levels in women or those under the age of 50 years [21]. This difference may be explained by the smoking status in the current analysis. We found that the circulating 25(OH)D levels in smokers are statistically significant lower than in non-smokers, and smoking status is associated with increased risk of NSCLC, especially in concentration of plasma 25(OH)D < 20 ng/mL. These findings stress the possibility that the increased NSCLC risk in smokers may be due to its influence on vitamin D. As we know, vitamin D has anti-inflammatory effects and promotes mechanisms of host defense, while cigarette smoke is pro-inflammatory and weakens host defense [22]. Epidemiological studies suggest that non-smokers have higher levels of vitamin D than smokers [23,24,25]. Cigarette smoke impairs mucociliary clearance and further damages the integrity of the respiratory epithelium [26]. Cigarette smoke has also been indicated to inhibit effects of vitamin D on NF-κB signaling and produce pro-inflammatory mediators by activating epithelial cells [27]. Hansdottir et al. proved that cigarette smoke interferes with vitamin D metabolism in the lungs [28]. Moreover, their study showed the impact of cigarette smoke on autophagy in alveolar macrophages and on vitamin D metabolism in respiratory epithelial cells: Cigarette smoke attenuates conversion of 1,25(OH)_2_D from 25(OH)D in respiratory epithelial cells. Smokers have an increased number of autophagosomes but defective autophagosome function. Decreased local generation of active vitamin D and autophagy defects in alveolar macrophages may contribute to impaired host defense in smokers [28]. Additionally, cigarette smoke may affect expression levels of the vitamin D receptor [29]. Therefore, cigarette smoke decreases the production of 1,25(OH)_2_D in lung epithelial cells, which might be overcome with higher plasma 25(OH)D concentrations.

In the present study, we also explored the nominally significant relationships between genetic polymorphisms in the vitamin D metabolism and NSCLC risk. The 24-hydroxylase encoded by *CYP24A1* catalyzes the conversion of both 1,25(OH)_2_D and 25(OH)D into a series of 24- and 23-hydroxlated products targeted for excretion along well-established pathways culminating in the water-soluble biliary metabolite, a 26,23-lactone or calcitroic acid [30]. CYP27B1ase catalyzes the second 1α-hydroxylation of 25(OH)D to produce 1,25(OH)_2_D in some extra-renal tissues and the kidney. Therefore, the concentrations of 25(OH)D and 1,25(OH)_2_D in blood are tightly controlled through feedback regulation of its biosynthesis and catabolism by CYP24A1ase and CYP27B1ase, respectively. A large genome-wide association study (GWAS) did not identify a significant relationship between *CYP27B1* and concentrations of circulating 25(OH)D, but variation in *CYP24A1* was significantly associated with plasma 25(OH)D concentrations [31]. In the present study, an interaction between plasma 25(OH)D levels and *CYP24A1* polymorphism rs6068810 was found, while there was no relationship between rs10877012 and rs3782130 polymorphism in *CYP27B1* gene and 25(OH)D levels. Our observation is in agreement with the finding of GWAS [32,33]. However, rs3782130 was located in the *CYP27B1* promoters, which are important *cis*-acting elements that regulate gene expression. Moreover, SNP in the region can influence transcription and gene function, while our findings suggested that rs3782130 variants may not result in attenuation of enzymatic activity in a Chinese population. We also found no interactions between *CYP24A1* or *CYP27B1* and polymorphism plasma 25(OH)D levels after accounting for multiple comparisons.

The CYP24A1ase plays a vital role in the vitamin D pathway, specifically regulating the level of 1,25(OH)2D [34]. A number of clinical studies have indicated that *CYP24A1* is overexpressed in lung cancer patients compared with normal control tissues [35,36,37,38]. Moreover, Anderson et al. demonstrated in various cancer cell lines that the anti-proliferative activity of 1,25(OH)_2_D is inversely proportional to *CYP24A1* mRNA expression [39]. When testing all NSCLC cases, we identified a correlation between SNP rs6068816 in *CYP24A1* and NSCLC risk. We observed a potential 54% reduction in NSCLC risk concerning the homozygous in rs6068816 for *CYP24A1* polymorphism. The amino acid sequence of *CYP24A1* isn’t altered by rs6068816 owning to a synonymous polymorphism, while it may affect intron splicing. The SNPs are located in silencers or the enhancers of splicing regions, that can influence the efficiency of mRNA splicing, and which in turn have effects on phenotype of biologic activities. Another possible reason for an association between rs6068816 in *CYP24A1* and NSCLC risk is that *CYP24A1* can evade growth control. This might be attributed to *CYP24A1* being increased in NSCLC tumors. Several studies have already examined the gain of 20q in gastro-esophageal junction [40], colon [41], breast [42], prostate [43], head and neck [44] as well as lung tumors [45,46]. Kong et al. reported that *CYP24A1* can reduce the 25(OH)D level and potentially increase NSCLC risk in cases with kinds of *CYP24A1* polymorphisms [47]. rs6013897 was not tested in the present study due to it having been reported in the GWAS studies [31,48,49], which were associated with vitamin D insufficiency.

The obvious reductions of NSCLC risk of several *VDR* polymorphisms, including rs1544410 in *Bsm1* and rs731236 in *Taq1*, were observed in our study. It is worth noting that all the *VDR* genotypes were related to declined NSCLC risk. An increased risk comparing TT vs. CC in *Taq1* in our study is consistent with the results observed in previous studies [50,51,52]. On the other hand, there are a few other studies that have also found no association with increased risk [53,54]. A decreased NSCLC risk with *Bsm1* in our findings is consistent with the study of Heist et al. [55]. However, our study did not show other variants in *VDR* that were associated with an increased NSCLC risk.

Notably, we found that the *CYP24A1* rs6068816 and *VDR* rs1544410 were significantly associated with increased 25(OH)D concentration in cases but not in controls. Together with the relationships between NSCLC risk and variants in genes associated with *VDR* and *CYP24A1*, these results support the concept that NSCLC carcinogenesis may be influenced by the vitamin D axis, including the interaction between the different components of vitamin D, which includes circulating vitamin D, the *CYP24A1*, and the *VDR*. The reason why there is a discrepancy in these SNPs is unclear. However, these results suggest that CYP24A1 protein is rate limiting for the amount of local vitamin D in cancer tissues, and elevated expression is associated with an adverse prognosis in cancer. Moreover, by regulating the level of vitamin D, this enzyme plays a role in Ca^2+^ homeostasis. Because of its important role in vitamin D metabolism and blood Ca^2+^ homeostasis, trivial changes in vitamin D hydroxylation activity could alter disease pathogenesis and outcome. Additionally, it exerts its oncogenic activities through its expressed protein in various cancers [56,57]. There is growing evidence that vitamin D may reduce the risk of developing NSCLC and that higher levels of vitamin D may be associated with better prognosis and improved outcome. However, our study did not provide the evidences that higher 25(OH)D levels are related to declined NSCLC risk, which suggests vitamin D may play a different role in disease initiation compared to disease progression, and SNPs that predict disease outcomes may be different than those that predict disease risk. There are many reports suggesting that vitamin D can reduce tumor growth of lung cancer in vitro [58] and in vivo [59].

Meanwhile, we found that smokers with homozygous for the common allele of *CYP24A1* rs6068816, *VDR* rs10735810, and *VDR* rs1544410 who had plasma 25(OH)D of ≥20 ng/mL had a significantly reduced NSCLC risk compared to women with plasma 25(OH)D <20 ng/mL. However, results showed that smokers with mutated homozygous *CYP24A1* rs2181874 and *VDR* rs10735810 had significantly increased NSCLC risk. The joint effects between smoking and *CYP24A1* rs2181874, *CYP24A1* rs6068816, *VDR* rs10735810, and *VDR* rs1544410 suggest *CYP24A1* rs6068816 and *VDR* rs1544410 mutations may reduce NSCLC susceptibility, whereas *CYP24A1* rs2181874 and *VDR* rs10735810 mutations may increase NSCLC susceptibility, especially in the smoking population. However, the possibility of false-positive results in the reported two joint-association analyses should be considered. Firstly, we did not extrapolate other populations in other areas excluding the Yunnan district, so the results have some limitation. Second, the smoker sample size in our study is comparatively small, which may not have enough statistical power to explore the true association, especially for haplotype analysis. Thirdly, there is the possibility of passive smoking or second-hand smoking in the cases’ family. Notably, Asian never-smoking females have an elevated incidence of lung cancer and lung cancer-related death rates compared with the European population [60]. It has been speculated that the high rate of lung cancer in Asian never-smokers is due to environmental factors such as second-hand smoke or cooking style [61,62]. Exposure to these carcinogens would lead to increased oxidative damage and an increase in the G>T transversion mutation rate [63]. We did not survey the degree of second-hand smoke exposure in our never-smoking patients. This raises the possibility that secondary tobacco smoke could be a confounding factor in these patients. Additionally, we did not observe a smoker-like mutation signature in any of our never-smoker patients, suggesting that this confounder might not be significant for our conclusions. One would expect long-term quitters to have a mutational pattern similar to that of never-smokers, and short-term quitters to resemble that of current smokers. It is known that 5 to 9 years of smoking cessation can lower the risk of lung cancer [64]. Therefore, the never-smoker-like signature in these patients cannot be accounted for by having quit smoking a long time ago. This highlights the importance of checking the molecular signature of patients with lung cancer irrespective of smoking status.

We explored two rather common SNPs, rs4588 and rs7041 in *GC*. Previous lung cancer studies have reported various results [65,66]. In the current study, it is shown that slight increases in NSCLC risk with rs7041 polymorphisms for rs7041 was a common nonsynonymous SNP in the *GC* gene. Previous studies have investigated rs7041 in relation to prostate cancer [67,68], breast cancer [69], basal cell carcinomas [70] and colon [71]. Our study indicates that rs7041 may be related to reduced NSCLC risk, which was similar to one recent study [47].

Our findings did not show that the *GC* polymorphism is associated with 25(OH)D levels. Overall, our results support the hypothesis that NSCLC carcinogenesis might be affected by the vitamin D levels and the interaction between the genetic variation and cigarette smoke. Further studies with larger sample size on interactions with calcium, plasma 25(OH)D levels, and vitamin D intake should be evaluated in terms of cancer occurrence.

## 4. Materials and Methods

### 4.1. Case and Control Selection

Eligible patients diagnosed with histologically confirmed primary NSCLC (*n* = 426) were enrolled in our study. We used the seventh edition of the TNM Classification of Malignant Tumors published in 2009 [72]. All cases were randomly selected for the study at the Third Affiliated Hospital of Kunming Medical College (TAHKMC) during July 2013 to December 2014. Clinical information collected in our study includes disease stage, tumor size, histological type, and lymph node metastasis. There were 334 cases of adenocarcinoma, 59 of squamous cell carcinoma (SCC), 25 of adenosquamous carcinoma and 8 cases of carcinoid tumor. As regards to the degree of differentiation, 50 of the cases were well differentiated, 234 were moderately differentiated and 142 were poorly differentiated. A total of 175 cases presented lymph node metastasis. In terms of postoperative pathological classification, 98 patients had stage IA, 69 had stage IB, 51 had stage IIA, 49 had stage IIB, 134 had stage IIIA, 17 had stage IIIB and 8 had stage IV disease.

The control subjects (*n* = 445) were recruited from the health check-up in the same hospital during the same period as the cases were recruited. Eventually, the controls were individually frequency-matched 1:1 with the cases for age (±6.2 years), race, gender, and date of blood collection (±1 month). All subjects of cases and controls agreed to participate and gave written, informed consent, and completed an in-person interview executed by a trained assistant of the study using a structured questionnaire. The questionnaire elicits information on sociodemographic characteristics and a number of potential risks including cigarette use, alcohol use, regular exercise, drinking habits, as well as eating habits including intake of milk, egg, any kind of meat (pork, beef, game, chick, duck, fatty fish, fish liver oil), fresh fruit, green vegetables, tea, coffee, pickled products. Medical history, history of pulmonary disease, family history of lung cancer as well as other lifestyle behaviors were also considered. Exclusion criteria included symptomatic brain metastases, spinal cord compression, uncontrolled massive pleural effusion, and other chronic diseases including history of pulmonary disease, and preoperative radiotherapy or chemotherapy [53,73]. Subjects taking calcium and vitamin D supplements during the previous 6 months were also excluded from the study. Smoking status was categorized as never smoking, current smoking and past smoking based on two questions: “Have you smoked more than 100 cigarettes in your life?” and “I used to smoke until the age of …”. Current smoking was defined as smoking either daily or less than daily (occasionally) up to his/her current age and having smoked more than 100 cigarettes in a lifetime. Past smoking was defined as having smoked more than 100 cigarettes and having stopped smoking for at least one year. Quit ratio was calculated as the ratio of the number of past smokers over the number of ever-smokers, i.e., total of current and past smokers [74].

### 4.2. Vitamin D Assay

Non-fasting baseline blood specimens were collected at the clinical centers. Quantification of plasma 25(OH)D was tested by radioimmunoassay (RIA) method (DiaSorin, Stillwater, MN, USA) [75]. Cases and controls were inspected continuously within batches. Details of the plasma 25(OH)D measurements have been described previously [12]. Assays were run in two batches; Quality controls (QC) were utilized to assess inter-assay accuracy and precision. During each run, QC samples (*n* = 5) were run together with the study samples. QC samples (*n* = 2; 17.3 and 50.4 ng/mL), pooled plasma samples (*n* = 1; 23.6 ng/mL) and commercially available external QC samples (*n* = 2; 63.9 and 107.9 ng/mL) provided by DiaSorin. The overall coefficients of variation (CVs) from blinded replicate QC samples in each batch were 9.6% and 5.8%. Laboratory staffs have been blinded to QC and control-case status.

### 4.3. SNP Selection and Genotyping Analysis

We used the following criteria to select a set of multi-population tag single-nucleotide polymorphisms (SNPs): (1) potentially functional, i.e. located in the 5′ flanking regions; the base pair change needs to be in predicted regulatory sequences in a promoter; 5′ untranslated (UTR) or 3′ untranslated (UTR) region could affect splicing or code regions with amino acid substitution or frequently studied variants from previous reports [76]; (2) minor allele frequencies of at least 5% and an r^2^ ≥ 80% were selected; and (3) related to plasma vitamin D levels in genome-wide association studies [77,78]. The 15 SNPs for genotyping were selected. They included five SNPs in *VDR*: *Bsm1* (rs1544410), *Taq1* (rs731236), *Apa1* (rs7975232), *Fok1* (rs10735810), *Cdx2* (rs11568820); six SNPs in 24-hydroxylase (*CYP24A1*): rs6068816, rs2244719, rs4809960, rs2762939, rs2181874, and rs2296241; two SNPs in the vitamin D-binding protein (*GC*): rs7041 and rs4588; and two SNPs in 1α-hydroxylase (*CYP27B1*): rs10877012 and rs3782130. Assays at all sites included at least two negative controls and replicate quality control samples (5% samples) per genotyping plates. The concordance rate between duplicate DNA samples ranged from 92% to 100% and completion rates ranged from 98% to 100%. The genotype frequencies among controls did not differ from the expected Hardy-Weinberg equilibrium proportions (*p* > 0.05).

Genomic DNA was extracted from fresh or frozen whole blood using a commercially available FlexiGen DNA isolation kit (Qiagen, Valencia, CA, USA) [79]. The PCR-RFLP method was used for genotyping each polymorphism. The sequences of the forward and reverse primers, the PCR conditions and characteristics of the SNPs used are summarized in Supplementary Materials Table S1 and Figure S2. Laboratory staff were blinded to the case-control status of the samples.

### 4.4. Statistical Analysis

Chi-square test was used to compare the frequency distributions between cases and controls of demographic variable, environmental factors and gene polymorphisms. For continuous variables, differences were compared by analysis of variance (ANOVA) or Student *t*-tests. The deviation from Hardy-Weinberg equilibrium (HWE) in controls and cases for each polymorphism were tested in our study. The unconditional logistic regression model was used to evaluate the connections between SNPs and NSCLC risk. In logistic regression analysis, odds ratios (ORs) and 95% confidence intervals (CIs) were calculated with adjustment for confounding factors. Subgroup analyses were also performed for each polymorphism by smoking status. The log-transformed plasma 25(OH)D concentrations were used to normalize the distribution of 25(OH)D. The multiple testing was performed by the Bonferroni method. All reported *p* values are two-sided. Results were considered statistically significant when the *p* value was smaller than 0.05. To assess the genotype score, we selected the SNP with the strongest evidence for association at each locus. We determined the inheritance model (dominant, recessive, or additive) for each individual SNP by analyzing the β-coefficients (log [odds ratio (OR)]) of the genotypes as categorical variables in logistic regression models adjusted for age, gender, and family history of NSCLC, and BMI (Table 3). In the dominant model, heterozygous genotypes were recoded as homozygous risk genotypes; in the recessive model, heterozygous genotypes were recoded as homozygous non-risk, whereas we coded genotypes as 0 for the non-risk homozygous genotype, 1 for the heterozygous genotype, and 2 for the homozygous risk genotype. In the additive model, the risk scores remained unchanged [80]. All statistical analyses were conducted using the SPSS statistical package, version 12.0 (IBM SPSS Inc., Chicago, IL, USA) and Stata/SE 11.0 (StataCorp, College Station, TX, USA).

## 5. Conclusions

In conclusion, we did not find that higher, prediagnostic levels of either vitamin D metabolite were associated with lower risk of NSCLC in Han Chinese. However, cigarette smoking may lead to the deficiency of vitamin D. Because vitamin D plays an important role in calcium absorption and bone health, cigarette smokers should increase their vitamin D intake. Furthermore, the specific vitamin D-related SNPs were associated with NSCLC risk, which supports the biologic explanation of a connection between the vitamin D pathway and NSCLC risk in a portion of the population with underlying genetic susceptibility. The relationship between NSCLC and circulating 25(OH)D should be explored in larger populations and different races in China. Prospective studies of long-term sun exposure could also be valuable in clarifying the role of vitamin D in NSCLC risk, although there are certainly substantial difficulties in estimating such exposures. Lastly, further investigations are necessary to replicate this finding and explore biological underpinnings of the plausibility of a gene-environment interaction, such as sun exposure, as it may yield additional insight into the association of vitamin D and NSCLC risk.

## Figures and Tables

**Table 1:** Descriptive characteristics of non-small cell lung cancer (NSCLC).

Characteristics	Cases (*n* = 426)	Controls (*n* = 445)	*p*
Age, years			0.283
Mean ± SD	57.4 ± 5.8	59.6 ± 4.7	
Gender (%)			0.447
Male	242 (56.8)	269 (60.4)	
Female	184 (43.2)	176 (39.6)	
Races (Han, %)	426 (100)	445 (100)	1
Married (%)			0.057
Yes	374 (87.8)	401 (90.1)	
No	52 (12.2)	44 (9.9)	
BMI, kg/m^2^ (% ^a^)			0.2
<25.0	94 (22.4)	65 (14.8)	
≥25.0	325 (77.6)	375 (85.2)	
Missing ^b^	7	5	
Family history of lung cancer (% ^a^)			<0.001
Yes	74 (17.8)	41 (9.5)	
No	341 (82.2)	390 (90.5)	
Missing ^b^	11	14	
Education (% ^a^)			
Less than high school	82 (19.5)	65 (14.8)	
High school graduate	339 (80.5)	373 (85.2)	
Missing ^b^	5	7	
Leisure physical activity (% ^a^)			0.252
<3 times/week	89 (21.2)	111 (25.6)	
≥3 times/week	330 (78.8)	322 (74.4)	
Missing ^b^	7	12	
Dietary vitamin D intake (μg/day)	5.7 ± 2.4	5.4 ± 3.1	0.759
Smoking history (% ^a^)			
Yes	297 (70.0)	214 (48.5)	0.001
No	127 (30.0)	227 (51.5)	
Missing ^b^	2	4	
Alcohol use (% ^a^)			0.132
Yes	261 (61.6)	275 (63.2)	
No	163 (38.4)	160 (36.8)	
Missing ^b^	2	10	
Plasma 25(OH)D (ng/mL)			0.251
Mean ± SD	21.0 ± 6.3	22.5 ± 7.3	

^a^ Percent of non-missing total; ^b^ Missing total not included in the percent distribution calculation; Standard Deviation (SD); body mass index (BMI); 25-hydroxy vitamin D [25(OH)D].

**Table 2:** Odds ratios (ORs) and 95% confidence intervals (CIs) for associations between the smoking and NSCLC risk was stratified by 25(OH)D.

	Concentration of Plasma 25(OH)D ^a,b^			
	<20 ng/mL		≥20 ng/mL	
Smoking Status	Cases/Controls	OR ^c^ (95% CI)	Cases/Controls	OR ^b,c^ (95% CI)
Non-smoker	85/149	1.00 (ref)	42/78	1.00 (ref)
Smoker	174/117	2.74 (1.97–3.01)	123/97	2.48 (2.05–2.97)
*p*		<0.001		<0.001

^a^ Missing value = 2 in case groups, Missing value = 4 in control groups; ^b^ Vitamin D deficiency was defined as 25(OH)D < 20 ng/mL; vitamin D sufficiency was defined as 25(OH)D ≥ 20ng/mL; ^c^ Covariates used for adjustment included age, gender, and family history NSCLC, and BMI.

**Table 3:** OR and 95% CIs for associations between vitamin D-related genotypes and NSCLC risk.

Gene (Reference SNP number, rs)	Genotype	Cases (*n* = 426)	Controls (*n* = 445)	OR ^a^ (95% CI)	*p*
*VDR* (rs10735810)	CC	166 (39.0%)	160 (36.0%)	1.00 (ref)	0.47
	CT	192 (45.1%)	204 (45.8%)	0.97 (0.73–1.41)	
	TT	68 (15.9%)	81 (18.2%)	1.15 (0.84–1.37)	
	TT + CT	260 (61.0%)	285 (64.0%)	1.09 (0.81–1.44)	
*VDR* (rs11568820)	TT	63 (14.8%)	52 (11.7%)	1.00 (ref)	0.25
	TC	324 (76.1%)	360 (80.9%)	1.22 (0.85–1.47)	
	CC	39 (9.1%)	33 (7.4%)	0.96 (0.79–1.29)	
	CC + TC	363 (85.2%)	393 (88.3%)	1.12 (0.87–1.54)	
*VDR* (rs1544410)	GG	403 (94.6%)	373 (83.8%)	1.00 (ref)	0.032
	GA	17 (4.0%)	49 (11.0%)	0.79 (0.64–1.13)	
	AA	6 (1.4%)	23 (5.2%)	0.78 (0.68–0.96)	
	AA + GA	23 (5.4%)	72 (16.2%)	0.78 (0.65–1.07)	
*VDR* (rs7975232)	CC	140 (32.9%)	142 (31.9%)	1.00 (ref)	0.76
	CA	191 (44.8%)	214 (48.1%)	1.11 (0.84–1.47)	
	AA	95 (22.3%)	89 (20.0%)	0.99 (0.63–1.35)	
	AA + CA	286 (67.1%)	303 (68.1)	1.02 (0.75–1.39)	
*VDR* (rs731236)	TT	409 (96.0%)	416 (93.5%)	1.00 (ref)	0.037
	TC	14 (3.3%)	27 (6.1%)	0.77 (0.59–0.99)	
	CC	3 (0.7%)	2 (0.4%)	0.84 (0.57–0.95)	
	CC + TC	17 (4.0%)	29 (6.5%)	0.79 (0.60–0.91)	
*CYP24A1* (rs6068816)	CC	170 (39.9%)	76 (17.1%)	1.00 (ref)	<0.001
	CT	222 (52.1%)	307 (69.0%)	0.85 (0.67–1.32)	
	TT	34 (8.0%)	62 (13.9%)	0.31 (0.21–0.47)	
	TT + CT	256 (60.1%)	369 (82.9%)	0.72 (0.58–1.02)	
*CYP24A1* (rs2244719)	TT	312 (73.2%)	320 (71.9%)	1.00 (ref)	0.65
	TC	89 (20.9%)	87 (19.6%)	1.10 (0.92–1.35)	
	CC	25 (5.9%)	38 (8.5%)	0.95 (0.77–1.16)	
	CC + TC	114 (26.8%)	125 (28.1%)	1.04 (0.88–1.23)	
*CYP24A1* (rs4809960)	TT	349 (81.9%)	360 (80.9%)	1.00 (ref)	0.14
	TC	62 (14.6%)	75 (16.9%)	0.85 (0.74–0.99)	
	CC	15 (3.5%)	10 (2.2%)	1.09 (0.78–1.52)	
	CC + TC	77 (18.1%)	85 (19.1%)	0.97 (0.75–1.21)	
*CYP24A1* (rs2762939)	GG	160 (37.5%)	156 (35.1%)	1.00 (ref)	0.50
	GC	192 (45.1%)	220 (49.4%)	1.09 (0.85–1.32)	
	CC	74 (17.4%)	69 (15.5%)	1.06 (0.79–1.33)	
	CC + GC	266 (62.5%)	289 (64.9%)	1.08 (0.73–1.25)	
*CYP24A1* (rs2181874)	GG	303 (71.1%)	340 (76.4%)	1.00 (ref)	0.07
	GA	84 (19.7%)	76 (17.1%)	1.17 (0.92–1.37)	
	AA	39 (9.2%)	29 (6.5%)	1.40 (0.85–1.92)	
	AA + GA	123 (28.9%)	105 (23.6%)	1.33 (0.89–1.65)	
*CYP24A1* (rs2296241)	GG	119 (27.9%)	114 (25.6%)	1.00 (ref)	0.09
	GA	230 (54.0%)	227 (51.0%)	1.30 (0.88–1.63)	
	AA	77 (18.1%)	104 (23.4%)	1.27 (0.76–1.55)	
	AA + GA	307 (72.1%)	331 (74.4%)	1.29 (0.92–1.72)	
*CYP27B1* (rs10877012)	GG	165 (38.7%)	160 (35.9%)	1.00 (ref)	0.37
	GT	209 (49.1%)	209 (47.0%)	1.10 (0.88–1.35)	
	TT	52 (12.2%)	76 (17.1%)	0.95 (0.76–1.21)	
	TT + GT	261 (61.3%)	285 (64.1%)	1.01 (0.75–1.32)	
*CYP27B1* (rs3782130)	CC	194 (45.5%)	187 (42.1%)	1.00 (ref)	0.15
	CG	149 (35.0%)	163 (36.6%)	0.82 (0.76–1.45)	
	GG	83 (19.5%)	95 (21.3%)	1.03 (0.89–1.34)	
	GG + CG	232 (54.5%)	258 (57.9%)	0.94 (0.78–1.38)	
*GC* (rs7041)	TT	175 (41.1%)	173 (38.8%)	1.00 (ref)	<0.001
	TG	230 (54.0%)	225 (50.6%)	0.79 (0.44–0.97)	
	GG	21 (4.9%)	47 (10.6%)	0.61 (0.41–0.93)	
	GG + TG	251 (58.9%)	272 (61.2%)	0.69 (0.38–1.15)	
*GC* (rs4588)	CC	230 (54.0%)	235 (52.8%)	1.00 (ref)	0.58
	CA	170 (39.9%)	173 (38.9%)	1.14 (0.91–1.45)	
	AA	26 (6.1%)	37 (8.3%)	0.93 (0.71–1.43)	
	AA + CA	196 (46.0%)	210 (47.2%)	1.11 (0.93–1.28)	

^a^ Covariates used for adjustment included age, gender, and family history of NSCLC and BMI.

**Table 4:** Comparison of plasma 25(OH)D concentrations (ng/mL) by polymorphisms in the vitamin D-related gene.

Gene (rs) Case/Control	Homozygous Common Allele (Mean ± SD)	Heterozygous (Mean ± SD)	Homozygous Minor Allele (Mean ± SD)	Heterozygous + Homozygous Minor Allele (Mean ± SD)	*p* ^a^	*p* ^b^
*VDR* (rs10735810)	CC	CT	TT	TT + CT		
Cases	21.5 ± 6.7	22.6 ± 7.7	20.6 ± 8.5	22.0 ± 7.1	0.56	0.67
Controls	26.1 ± 5.4	25.3 ± 6.7	24.5 ± 8.2	24.9 ± 7.8	0.55	0.47
*VDR* (rs11568820)	TT	TC	CC	CC + TC		
Cases	27.0 ± 6.4	24.9 ± 8.4	27.4 ± 7.4	25.9 ± 8.9	0.70	0.45
Controls	25.7 ± 9.2	27.4 ± 8.0	27.1 ± 10.3	27.3 ± 7.4	0.56	0.87
*VDR* (rs1544410)	GG	GA	AA	AA + GA		
Cases	16.5 ± 8.6	20.7 ± 6.8	25.2 ± 7.3	24.6 ± 7.4	0.009	0.008
Controls	21.7 ± 4.6	23.0 ± 7.1	23.4 ± 7.0	23.1 ± 6.9	0.23	0.39
*VDR* (rs7975232)	CC	CA	AA	AA + CA		
Cases	26.7 ± 6.7	25.9 ± 5.1	28.1 ± 7.7	26.9 ± 8.0	0.89	0.87
Controls	28.9 ± 5.7	25.8 ± 7.9	27.9 ± 7.3	27.1 ± 8.8	0.65	0.31
*VDR* (rs731236)	TT	TC	CC	CC + TC		
Cases	24.5 ± 6.8	24.5 ± 6.9	23.4 ± 6.1	24.5 ± 5.4	0.70	0.87
Controls	27.7 ± 9.8	26.8 ± 5.1	28.6 ± 6.4	27.6 ± 7.5	0.80	0.65
*CYP24A1* (rs6068816)	CC	CT	TT	TT + CT		
Cases	16.4 ± 4.5	20.7 ± 7.3	25.7 ± 10.3	23.8 ± 7.7	0.007	0.01
Controls	17.9 ± 5.2	18.9 ± 7.8	18.4 ± 7.1	18.5 ± 7.2	0.20	0.27
*CYP24A1* (rs2244719)	TT	TC	CC	CC + TC		
Cases	28.6 ± 8.2	27.6 ± 7.8	29.0 ± 6.9	28.3 ± 6.2	0.80	0.85
Controls	26.8 ± 7.2	27.4 ± 6.5	25.5 ± 6.3	27.2 ± 6.3	0.51	0.44
*CYP24A1* (rs4809960)	TT	TC	CC	CC + TC		
Cases	24.6 ± 6.1	26.8 ± 8.2	25.7 ± 7.7	27.6 ± 7.4	0.76	0.60
Controls	25.9 ± 6.5	26.5 ± 7.6	27.7 ± 8.6	27.0 ± 8.0	0.62	0.56
*CYP24A1* (rs2762939)	GG	GC	CC	CC + GC		
Cases	25.9 ± 4.8	27.9 ± 7.1	27.1 ± 8.9	27.8 ± 7.0	0.61	0.70
Controls	31.4 ± 10.1	29.6 ± 8.9	29.7 ± 11.6	29.0 ± 10.5	0.90	0.93
*CYP24A1* (rs2181874)	GG	GA	AA	AA + GA		
Cases	26.2 ± 6.1	24.4 ± 5.7	24.4 ± 7.8	24.5 ± 7.6	0.29	0.15
Controls	28.1 ± 5.7	26.7 ± 8.9	24.9 ± 5.7	25.4 ± 8.1	0.13	0.09
*CYP24A1* (rs2296241)	GG	GA	AA	AA + GA		
Cases	24.6 ± 9.6	21.9 ± 9.5	20.5 ± 8.7	21.5 ± 8.5	0.13	0.21
Controls	25.7 ± 11.2	23.4 ± 10.1	24.0 ± 10.4	24.9 ± 9.5	0.36	0.61
*CYP27B1* (rs10877012)	GG	GT	TT	TT + GT		
Cases	28.1 ± 7.7	28.0 ± 8.3	29.4 ± 10.1	28.6 ± 9.8	0.80	0.71
Controls	27.1 ± 8.3	27.9 ± 4.9	29.9 ± 8.7	29.1 ± 5.6	0.33	0.34
*CYP27B1* (rs3782130)	CC	CG	GG	GG + CG		
Cases	27.0 ± 6.7	27.8 ± 8.9	25.7 ± 6.1	26.4 ± 7.4	0.81	0.77
Controls	26.5 ± 8.1	28.5 ± 14.3	28.2 ± 8.6	28.6 ± 10.7	0.25	0.10
*GC* (rs7041)	TT	TG	GG	GG + TG		
Cases	24.7 ± 8.0	25.6 ± 6.9	26.1 ± 7.3	26.0 ± 6.3	0.37	0.43
Controls	28.0 ± 10.2	27.8 ± 7.1	29.5 ± 7.3	29.1 ± 8.4	0.87	0.61
*GC* (rs4588)	CC	CA	AA	AA + CA		
Cases	24.7 ± 7.1	26.7 ± 7.9	27.1 ± 6.9	26.9 ± 7.1	0.73	0.74
Controls	27.2 ± 7.1	27.0 ± 7.5	29.1 ± 10.8	28.0 ± 6.5	0.19	0.41

^a^ Comparing across all three genotypes; ^b^ Comparing homozygous major genotype to the combination of heterozygous and homozygous minor genotypes.

**Table 5:** ORs and 95% CIs for the joint association between vitamin D-related polymorphisms and plasma 25(OH)D on NSCLC risk.

		Homozygous Common Allele		Heterozygous and Homozygous Minor Allele		
Polymorphism	Plasma 25(OH)D (ng/mL) ^a^	Cases/Controls	OR (95% CI)	Cases/Controls	OR (95% CI)	*p*-Interaction ^b^
*VDR* (rs10735810)	<20 ng/mL	47/38	1.00 (ref)	78/56	1.00 (ref)	0.015
	≥20 ng/mL	119/122	0.47 (0.24–1.43)	182/229	0.46 (0.31–0.63)	
*VDR* (rs11568820)	<20 ng/mL	16/11	1.00 (ref)	109/81	1.00(ref)	0.263
	≥20 ng/mL	47/41	0.66 (0.34–1.52)	254/312	0.68 (0.47–0.95)	
*VDR* (rs1544410)	<20 ng/mL	123/81	1.00 (ref)	6/15	1.00 (ref)	0.021
	≥20 ng/mL	280/292	0.73 (0.44–1.07)	17/57	0.52 (0.29–0.97)	
*VDR* (rs7975232)	<20 ng/mL	42/29	1.00 (ref)	84/64	1.00 (ref)	0.631
	≥20 ng/mL	98/113	0.69 (0.41–1.21)	202/239	0.74 (0.59–1.32)	
*VDR* (rs731236)	<20 ng/mL	124/83	1.00 (ref)	5/6	1.00 (ref)	0.841
	≥20 ng/mL	285/333	0.55 (0.27–0.66)	12/23	0.60 (0.39–0.88)	
*CYP24A1* (rs6068816)	<20 ng/mL	49/16	1.00 (ref)	82/79	1.00 (ref)	0.002
	≥20 ng/mL	121/60	0.41(0.28–0.61)	174/290	0.48(0.25–0.65)	
*CYP24A1* (rs2244719)	<20 ng/mL	87/66	1.00 (ref)	35/26	1.00 (ref)	0.731
	≥20 ng/mL	225/254	0.65 (0.42–0.91)	79/99	0.78 (0.49–0.99)	
*CYP24A1* (rs4809960)	<20ng/mL	100/71	1.00 (ref)	23/18	1.00 (ref)	0.724
	≥20 ng/mL	249/289	0.58 (0.31–1.05)	54/67	0.67 (0.45–0.88)	
*CYP24A1* (rs2762939)	<20 ng/mL	47/33	1.00 (ref)	78/61	1.00 (ref)	0.691
	≥20 ng/mL	113/123	0.67 (0.49–0.98)	188/228	0.53 (0.31–0.87)	
*CYP24A1* (rs2181874)	<20 ng/mL	98/77	1.00 (ref)	46/21	1.00 (ref)	0.113
	≥20 ng/mL	205/263	0.47 (0.28–0.90)	77/84	0.72 (0.58–0.93)	
*CYP24A1* (rs2296241)	<20 ng/mL	33/23	1.00 (ref)	95/73	1.00 (ref)	0.832
	≥20 ng/mL	86/91	0.79 (0.58–1.09)	212/258	0.61 (0.40–0.97)	
*CYP27B1* (rs10877012)	<20 ng/mL	48/32	1.00 (ref)	78/64	1.00 (ref)	0.875
	≥20 ng/mL	117/128	0.71 (0.53–0.98)	183/221	0.74 (0.49–1.14)	
*CYP27B1* (rs3782130)	<20 ng/mL	53/28	1.00 (ref)	70/62	1.00 (ref)	0.962
	≥20 ng/mL	141/159	0.80 (0.69–1.24)	162/196	0.73 (0.53–1.00)	
*GC* (rs7041)	<20 ng/mL	43/36	1.00 (ref)	73/59	1.00 (ref)	0.182
	≥20 ng/mL	132/137	0.95 (0.81–1.53)	178/213	0.57 (0.41–0.82)	
*GC* (rs4588)	<20 ng/mL	61/43	1.00 (ref)	64/51	1.00 (ref)	0.804
	≥20 ng/mL	169/192	0.52 (0.31–0.90)	132/159	0.71 (0.39–0.86)	

^a^ Vitamin D deficiency was defined as 25(OH)D <20 ng/mL; vitamin D sufficiency was defined as 25(OH)D ≥20 ng/mL; ^b^ Interaction is between plasma 25(OH)D and the homozygous common allele and heterozygous or homozygous minor allele, with <20 ng/mL as the reference.

**Table 6:** Comparison of plasma 25(OH)D concentrations (ng/mL) in smokers and non-smokers with different polymorphisms.

Gene (rs) Case/Control Smoking Status	Homozygous Common Allele (Mean ± SD)	Heterozygous (Mean ± SD)	Homozygous Minor Allele (Mean ± SD)	Heterozygous + Homozygous Minor Allele (Mean ± SD)	*p* ^a^	*p* ^b^
*VDR* (rs10735810)	CC	CT	TT	TT + CT		
Cases						
Smoker	19.4 ± 7.8	16.9 ± 6.8	17.8 ± 5.9	17.5 ± 6.0	0.67	0.32
Non-smoker	24.8 ± 4.5	26.4 ± 10.6	21.8 ± 7.2	23.6 ± 8.4	0.45	0.87
Controls						
Smoker	26.8 ± 3.5	24.7 ± 5.7	23.6 ± 5.4	24.1 ± 5.4	0.78	0.65
Non-smoker	25.8 ± 6.4	28.6 ± 9.3	24.6 ± 7.1	26.8 ± 8.2	0.41	0.44
*VDR* (rs11568820)	TT	TC	CC	CC + TC		
Cases						
Smoker	26.7 ± 7.8	25.8 ± 10.5	27.8 ± 10.2	26.6 ± 9.5	0.88	0.62
Non-smoker	27.8 ± 6.1	24.7 ± 7.9	26.3 ± 8.6	25.4 ± 7.2	0.75	0.37
Controls						
Smoker	24.7 ± 6.7	25.8 ± 7.8	27.4 ± 8.8	26.4 ± 5.5	0.53	0.78
Non-smoker	26.7 ± 7.8	28.5 ± 9.2	26.6 ± 9.1	27.6 ± 7.9	0.84	0.93
*VDR* (rs1544410)	GG	GA	AA	AA + GA		
Cases						
Smoker	14.5 ± 7.8	18.5 ± 9.6	25.6 ± 7.0	24.1 ± 8.8	0.005	0.003
Non-smoker	18.9 ± 10.7	21.6 ± 4.7	25.1 ± 7.9	24.7 ± 5.6	0.01	0.01
Controls						
Smoker	19.6 ± 12.5	22.4 ± 6.7	24.9 ± 7.8	23.8 ± 8.9	0.67	0.76
Non-smoker	22.3 ± 6.2	24.9 ± 7.5	25.4 ± 5.6	25.0 ± 7.5	0.08	0.12
*VDR* (rs7975232)	CC	CA	AA	AA + CA		
Cases						
Smoker	26.5 ± 12.2	25.7 ± 10.7	27.0 ± 5.6	26.3 ± 6.2	0.86	0.90
Non-smoker	28.9 ± 14.5	26.6 ± 6.2	28.4 ± 7.2	27.4 ± 7.3	0.91	0.76
Controls						
Smoker	26.5 ± 8.4	24.6 ± 6.8	27.4 ± 7.9	26.4 ± 8.5	0.78	0.86
Non-smoker	30.4 ± 12.5	26.7 ± 10.6	28.4 ± 6.8	27.7 ± 9.3	0.56	0.12
*VDR* (rs731236)	TT	TC	CC	CC + TC		
Cases						
Smoker	21.5 ± 7.5	23.5 ± 3.2	22.5 ± 6.9	23.1 ± 4.7	0.88	0.91
Non-smoker	26.6 ± 4.4	25.1 ± 7.2	25.5 ± 5.6	25.4 ± 6.8	0.69	0.70
Controls						
Smoker	29.0 ± 11.4	22.5 ± 5.6	29.3 ± 6.7	27.4 ± 10.2	0.87	0.75
Non-smoker	26.8 ± 9.7	28.3 ± 6.0	27.4 ± 7.1	27.9 ± 6.7	0.75	0.59
*CYP24A1* (rs6068816)	CC	CT	TT	TT + CT		
Cases						
Smoker	14.7 ± 5.2	16.3 ± 7.8	18.7 ± 5.8	17.4 ± 6.8	0.002	0.02
Non-smoker	18.4 ± 4.7	22.4 ± 6.9	27.2 ± 13.4	25.6 ± 9.2	0.009	0.003
Controls						
Smoker	15.6 ± 7.8	17.8 ± 10.3	16.5 ± 6.7	17.0 ± 7.8	0.18	0.12
Non-smoker	19.5 ± 6.7	18.6 ± 8.6	19.6 ± 6.9	19.2 ± 7.5	0.24	0.35
*CYP24A1* (rs2244719)	TT	TC	CC	CC + TC		
Cases						
Smoker	26.4 ± 7.9	27.6 ± 10.9	27.7 ± 6.8	27.6 ± 5.7	0.95	0.85
Non-smoker	29.2 ± 11.3	28.5 ± 9.8	29.6 ± 8.5	29.0 ± 8.7	0.75	0.84
Controls						
Smoker	27.0 ± 7.9	28.5 ± 5.7	24.6 ± 8.4	27.7 ± 6.8	0.54	0.74
Non-smoker	26.7 ± 9.1	25.7 ± 8.9	26.1 ± 6.7	26.0 ± 7.4	0.50	0.31
*CYP24A1* (rs4809960)	TT	TC	CC	CC + TC		
Cases						
Smoker	23.7 ± 5.8	25.6 ± 9.2	24.7 ± 7.1	25.5 ± 6.7	0.78	0.67
Non-smoker	25.5 ± 5.7	28.3 ± 7.6	27.6 ± 8.7	28.0 ± 8.4	0.62	0.56
Controls						
Smoker	26.8 ± 6.7	25.2 ± 6.5	29.7 ± 9.8	27.8 ± 6.5	0.87	0.67
Non-smoker	24.7 ± 7.8	27.8 ± 7.1	28.3 ± 10.3	28.1 ± 8.7	0.54	0.26
*CYP24A1* (rs2762939)	GG	GC	CC	CC + GC		
Cases						
Smoker	25.6 ± 4.6	26.4 ± 6.8	26.6 ± 9.1	26.7 ± 7.6	0.71	0.65
Non-smoker	26.8 ± 5.3	28.5 ± 7.9	27.8 ± 7.6	28.1 ± 7.7	0.56	0.73
Controls						
Smoker	30.3 ± 11.4	28.6 ± 9.5	29.8 ± 10.5	28.8 ± 9.6	0.91	0.97
Non-smoker	32.3 ± 14.5	30.4 ± 8.7	29.5 ± 12.1	29.9 ± 11.4	0.86	0.90
*CYP24A1* (rs2181874)	GG	GA	AA	AA + GA		
Cases						
Smoker	25.7 ± 6.7	24.2 ± 9.2	23.5 ± 10.4	23.1 ± 8.6	0.26	0.17
Non-smoker	27.6 ± 4.6	26.7 ± 7.9	25.2 ± 6.4	25.8 ± 6.8	0.31	0.12
Controls						
Smoker	28.0 ± 6.5	25.6 ± 7.8	24.6 ± 7.2	24.4 ± 6.5	0.06	0.07
Non-smoker	28.5 ± 7.9	27.8 ± 6.8	25.4 ± 7.0	26.4 ± 10.4	0.17	0.12
*CYP24A1* (rs2296241)	GG	GA	AA	AA + GA		
Cases						
Smoker	24.3 ± 11.3	21.6 ± 10.5	20.6 ± 9.6	21.4 ± 7.5	0.18	0.25
Non-smoker	24.8 ± 7.4	22.7 ± 9.8	20.5 ± 8.5	21.8 ± 9.4	0.06	0.17
Controls						
Smoker	24.4 ± 6.5	22.7 ± 8.3	23.5 ± 11.4	24.5 ± 10.7	0.44	0.77
Non-smoker	26.4 ± 14.7	25.7 ± 12.1	24.7 ± 10.9	25.1 ± 11.1	0.22	0.25
*CYP27B1* (rs10877012)	GG	GT	TT	TT + GT		
Cases						
Smoker	26.1 ± 9.5	28.6 ± 7.8	30.1 ± 10.3	28.4 ± 11.4	0.87	0.65
Non-smoker	28.8 ± 6.4	27.5 ± 8.9	29.7 ± 9.4	28.6 ± 9.1	0.76	0.86
Controls						
Smoker	27.0 ± 7.2	25.7 ± 8.9	29.7 ± 6.7	28.8 ± 8.1	0.21	0.45
Non-smoker	27.8 ± 9.8	29.9 ± 7.4	30.4 ± 7.9	30.0 ± 7.7	0.43	0.28
*CYP27B1* (rs3782130)	CC	CG	GG	GG + CG		
Cases						
Smoker	24.5 ± 5.6	26.5 ± 7.5	26.0 ± 5.8	26.3 ± 6.1	0.87	0.56
Non-smoker	27.5 ± 8.5	29.8 ± 10.6	28.6 ± 7.0	28.1 ± 9.2	0.73	0.96
Controls						
Smoker	25.4 ± 5.1	30.2 ± 11.5	27.5 ± 9.8	28.9 ± 10.8	0.13	0.09
Non-smoker	27.6 ± 9.6	27.7 ± 10.6	28.8 ± 7.9	28.1 ± 10.7	0.42	0.12
*GC* (rs7041)	TT	TG	GG	GG + TG		
Cases						
Smoker	23.9 ± 7.8	26.5 ± 6.7	25.3 ± 6.5	26.1 ± 5.9	0.45	0.34
Non-smoker	25.1 ± 8.3	24.7 ± 7.8	26.2 ± 5.8	25.7 ± 7.1	0.32	0.56
Controls						
Smoker	28.0 ± 11.8	28.9 ± 6.8	29.8 ± 7.9	29.3 ± 7.4	0.91	0.56
Non-smoker	28.1 ± 9.5	28.6 ± 9.4	29.6 ± 8.1	29.0 ± 9.2	0.83	0.67
*GC* (rs4588)	CC	CA	AA	AA + CA		
Cases						
Smoker	24.3 ± 6.1	26.3 ± 5.9	27.0 ± 8.3	26.7 ± 7.4	0.43	0.61
Non-smoker	25.1 ± 6.0	27.1 ± 9.4	27.4 ± 5.7	27.3 ± 8.7	0.85	0.93
Controls						
Smoker	25.7 ± 3.5	26.4 ± 5.6	27.4 ± 7.6	27.0 ± 6.3	0.21	0.34
Non-smoker	29.6 ± 8.2	27.3 ± 8.9	29.6 ± 11.5	28.5 ± 10.2	0.16	0.56

^a^ Comparing across all three genotypes; ^b^ Comparing the homozygous major genotype to the combination of heterozygous and homozygous minor genotypes.

**Table 7:** Associations between NSCLC risk and SNPs by smoking status.

Genotype	Non-Smoker			Smoker		
	Cases (%)	Controls (%)	OR ^a^ (95% CI)	Cases (%)	Controls (%)	OR ^a^ (95% CI)
*CYP24A1* (rs2181874)						
GG + GA	102 (80.3%)	215 (94.7%)	1.00 (ref)	256 (86.2%)	197 (92.1%)	1.00 (ref)
AA	25 (19.7%)	12 (5.3%)	2.14 (1.47–3.43)	41 (13.8%)	17 (7.9%)	3.57 (2.66–4.74)
*p*	0.031			0.019		
*p*-Interaction	0.016					
*CYP24A1* (rs6068816)						
CC + CT	91 (71.7%)	158 (74.0%)	1.00 (ref)	282 (94.9%)	182 (85.1%)	1.00 (ref)
TT	36 (28.3%)	69 (30.4%)	0.84 (0.65–1.41)	15 (5.1%)	32 (14.9%)	0.43 (0.27–1.02)
*p*	0.079			0.006		
*p*-Interaction	0.038					
*VDR* (rs10735810)						
CC + CT	112 (88.2%)	198 (87.2%)	1.00 (ref)	237 (79.8%)	186 (86.9%)	1.00 (ref)
TT	15 (11.8%)	29 (12.8%)	1.16 (0.82–1.34)	60 (20.2%)	28 (13.1%)	1.93 (1.41–2.76)
*p*	0.256			0.015		
*p*-Interaction	0.004					
*VDR* (rs1544410)						
GG + GA	116 (91.3%)	172 (75.8%)	1.00 (ref)	294 (98.9%)	175 (81.8%)	1.00 (ref)
AA	11 (8.7%)	55 (24.2%)	0.51 (0.34–1.17)	3 (1.0%)	39 (18.2%)	0.26 (0.20–0.69)
*p*	0.002			0.001		
*p*-Interaction	0.002					

^a^ Covariates used for adjustment included age, gender, and family history of NSCLC and BMI.

## Supplementary Materials

Supplementary materials can be found at www.mdpi.com/1422-0067/17/10/1597/s1.

## References

[1] Jemal A., Bray F., Center M.M., Ferlay J., Ward E., Forman D. (2011). Global cancer statistics. CA. Cancer J. Clin..

[2] De Groot P., Munden R.F. (2012). Lung cancer epidemiology, risk factors, and prevention. Radiol. Clin. N. Am..

[3] Ali M.M., Vaidya V. (2007). Vitamin D and cancer. J. Cancer Res. Ther..

[4] Mitchell D. (2011). The relationship between vitamin D and cancer. Clin. J. Oncol. Nurs..

[5] Trump D.L., Chadha M.K., Sunga A.Y., Fakih M.G., Ashraf U., Silliman C.G., Hollis B.W., Nesline M.K., Tian L., Tan W. (2009). Vitamin D deficiency and insufficiency among patients with prostate cancer. BJU Int..

[6] Hines S.L., Jorn H.K., Thompson K.M., Larson J.M. (2010). Breast cancer survivors and vitamin D: A review. Nutrition.

[7] LaPar D.J., Nagji A.S., Bhamidipati C.M., Kozower B.D., Lau C.L., Ailawadi G., Jones D.R. (2011). Seasonal variation influences outcomes following lung cancer resections. Eur. J. Cardiothorac. Surg..

[8] Holick M.F. (2009). Vitamin D status: Measurement, interpretation, and clinical application. Ann. Epidemiol..

[9] Jones G., Prosser D.E., Kaufmann M. (2012). 25-Hydroxyvitamin d-24-hydroxylase (*CYP24A1*): Its important role in the degradation of vitamin D. Arch. Biochem. Biophys..

[10] Li L.H., Yin X.Y., Wu X.H., Zhang L., Pan S.Y., Zheng Z.J., Wang J.G. (2014). Serum 25(OH)D and vitamin D status in relation to *VDR*, *GC* and *CYP2R1* variants in Chinese. Endocr. J..

[11] Speeckaert M., Huang G., Delanghe J.R., Taes Y.E. (2006). Biological and clinical aspects ofthe vitamin D binding protein (Gc-globulin) and its polymorphism. Clin. Chim. Acta.

[12] Mawer E.B., Hayes M.E., Heys S.E., Davies M., White A., Stewart M.F., Smith G.N. (1994). Constitutive synthesis of 1,25-dihydroxyvitamin D3 by a human small cell lung cancer cell line. J. Clin. Endocrinol. Metab..

[13] Zhou W., Heist R.S., Liu G., Asomaning K., Neuberg D.S., Hollis B.W., Wain J.C., Lynch T.J., Giovannucci E., Su L. (2007). Circulating 25-hydroxyvitamin D levels predict survival in early-stage non-small-cell lung cancer patients. J. Clin. Oncol..

[14] Liu Y., Chen W., Hu Z.B., Xu L., Shu Y.Q., Pan S.Y., Dai J.C., Jin G.F., Ma H.X., Shen H.B. (2011). Plasma vitamin D levels and vitamin D receptor polymorphisms are associated with survival of non-small cell lung cancer. Chin. J. Cancer Res..

[15] Yao S., Haddad S.A., Hu Q., Liu S., Lunetta K.L., Ruiz-Narvaez E.A., Hong C.C., Zhu Q., Sucheston-Campbell L., Cheng T.Y. (2016). Genetic variations in vitamin D-related pathways and breast cancer risk in African American women in the AMBER consortium. Int. J. Cancer.

[16] Arem H., Yu K., Xiong X., Moy K., Freedman N.D., Mayne S.T., Albanes D., Arslan A.A., Austin M., Bamlet W.R. (2015). Vitamin D metabolic pathway genes and pancreatic cancer risk. PLoS ONE.

[17] Verone-Boyle A.R., Shoemaker S., Attwood K., Morrison C.D., Makowski A.J., Battaglia S., Hershberger P.A. (2016). Diet-derived 25 hydroxyvitamin D3 activates vitamin D receptor target gene expression and suppresses EGFR mutant non-small cell lung cancer growth in vitro and in vivo. Oncotarget.

[18] Sharma K., Goehe R.W., Di X., Hicks M.A., Torti S.V., Torti F.M., Harada H., Gewirtz D.A. (2014). A novel cytostatic form of autophagy in sensitization of non-small cell lung cancer cells to radiation by vitamin D and the vitamin D analog, EB 1089. Autophagy.

[19] Turna A., Pekçolaklar A., Metin M., Yaylim I., Gurses A. (2012). The effect of season of operation on the survival of patients with resected non-small cell lung cancer. Interact. Cardiovasc. Thorac. Surg..

[20] Porojnicu A.C., Robsahm T.E., Dahlback A., Berg J.P., Christiani D., Bruland O.S., Moan J. (2007). Seasonal and geographical variations in lung cancer prognosis in Norway. Does vitamin D from the sun play a role?. Lung Cancer.

[21] Kilkkinen A., Knekt P., Heliövaara M., Rissanen H., Marniemi J., Hakulinen T., Aromaa A. (2008). Vitamin D status and the risk of lung cancer: A cohort study in Finland. Cancer Epidemiol. Biomark. Prev..

[22] Fletcher J. (2016). Vitamin D deficiency in patients with inflammatory bowel disease. Br. J. Nurs..

[23] Brot C., Jorgensen N.R., Sorensen O.H. (1999). The influence of smoking on vitamin D status and calcium metabolism. Eur. J. Clin. Nutr..

[24] Jorde R., Saleh F., Figenschau Y., Kamycheva E., Haug E., Sundsfjord J. (2005). Serum parathyroid hormone (PTH) levels in smokers and non- smokers. The fifth Tromso study. Eur. J. Endocrinol..

[25] Need A.G., Kemp A., Giles N., Morris H.A., Horowitz M., Nordin B.E. (2002). Relationships between intestinal calcium absorption, serum vitamin D metabolites and smoking in postmenopausal women. Osteoporos. Int..

[26] Stampfli M.R., Anderson G.P. (2009). How cigarette smoke skews immune responses to promote infection, lung disease and cancer. Nat. Rev. Immunol..

[27] Mio T., Romberger D.J., Thompson A.B., Robbins R.A., Heires A., Rennard S.I. (1997). Cigarette smoke induces interleukin-8 release from human bronchial epithelial cells. Am. J. Respir. Crit. Care Med..

[28] Hansdottir S. (2011). Modulation of Lung Innate Immunity by Vitamin D and Cigarette Smoke. Ph.D. Thesis.

[29] Haley K.J., Manoli S.E., Tantisira K.G., Litonjua A.A., Nguyen P., Kobzik L., Weiss S.T. (2009). Maternal smoking causes abnormal expression of the vitamin D receptor. Am. J. Respir. Crit. Care Med..

[30] Jones G., Prosser D.E., Kaufmann M. (2014). Cytochrome P450-mediated metabolism of vitamin D. J. Lipid Res..

[31] Wang T.J., Zhang F., Richards J.B., Kestenbaum B., van Meurs J.B., Berry D., Kiel D.P., Streeten E.A., Ohlsson C., Koller D.L. (2010). Common genetic determinants of vitamin D insufficiency: A genome-wide association study. Lancet.

[32] Joshi A.D., Andersson C., Buch S., Stender S., Noordam R., Weng L.C., Weeke P.E., Auer P.L., Boehm B., Chen C. (2016). Four susceptibility loci for gallstone disease identified in a meta-analysis of genome-wide association studies. Gastroenterology.

[33] Zhu M., Cheng Y., Dai J., Xie L., Jin G., Ma H., Hu Z., Shi Y., Lin D., Shen H. (2015). Genome-wide association study based risk prediction model in predicting lung cancer risk in Chinese. Zhonghua Liu Xing Bing Xue Za Zhi.

[34] Cargill M., Altshuler D., Ireland J., Sklar P., Ardlie K., Patil N., Shaw N., Lane C.R., Lim E.P., Kalyanaraman N. (1999). Characterization of single-nucleotide polymorphisms in coding regions of human genes. Nat. Genet..

[35] Chen G., Kim S.H., King A.N., Zhao L., Simpson R.U., Christensen P.J., Wang Z., Thomas D.G., Giordano T.J., Lin L. (2011). *CYP24A1* is an independent prognostic marker of survival in patients with lung adenocarcinoma. Clin. Cancer Res..

[36] Ramnath N., Nadal E., Jeon C.K., Sandoval J., Colacino J., Rozek L.S., Christensen P.J., Esteller M., Beer D.G., Kim S.H. (2014). Epigenetic regulation of vitamin D metabolism in human lung adenocarcinoma. J. Thorac. Oncol..

[37] Zhang Q., Kanterewicz B., Buch S., Petkovich M., Parise R., Beumer J., Lin Y., Diergaarde B., Hershberger P.A. (2012). *CYP24* inhibition preserves 1α,25-dihydroxyvitamin D(3) anti-proliferative signaling in lung cancer cells. Mol. Cell. Endocrinol..

[38] Jones G., Ramshaw H., Zhang A., Cook R., Byford V., White J., Petkovich M. (1999). Expression and activity of vitamin D-metabolizing cytochrome P450s (CYP1alpha and CYP24) in human nonsmall cell lung carcinomas. Endocrinology.

[39] Anderson M.G., Nakane M., Ruan X., Kroeger P.E., Wu-Wong J.R. (2006). Expression of *VDR* and *CYP24A1* mRNA in human tumors. Cancer Chemother. Pharmacol..

[40] Zhou L., Zhang X., Chen X., Liu L., Lu C., Tang X., Shi J., Li M., Zhou M., Zhang Z. (2012). GC Glu416Asp and Thr420Lys polymorphisms contribute to gastrointestinal cancer susceptibility in a Chinese population. Int. J. Clin. Exp. Med..

[41] Hummel D.M., Fetahu I.S., Gröschel C., Manhardt T., Kállay E. (2014). Role of proinflammatory cytokines on expression of vitamin D metabolism and target genes incolon cancer cells. J. Steroid Biochem. Mol. Biol..

[42] Shui I.M., Mondul A.M., Lindström S., Tsilidis K.K., Travism R.C., Gerke T., Albanes D., Mucci L.A., Giovannucci E., Kraft P. (2015). Breast and prostate cancer cohort consortium group: Circulating vitamin D, vitamin D-related genetic variation, and risk of fatal prostate cancer in the national cancer institute breast and prostate cancer cohort consortium. Cancer.

[43] Gilbert R., Bonilla C., Metcalfe C., Lewis S., Evans D.M., Fraser W.D., Kemp J.P., Donovan J.L., Hamdy F.C., Neal D.E. (2015). Associations of vitamin D pathway genes with circulating 25-hydroxyvitamin-D, 1,25-dihydroxyvitamin-D, and prostate cancer: A nested case-control study. Cancer Causes Control.

[44] Azad A.K., Bairati I., Qiu X., Huang H., Cheng D., Liu G., Meyer F., Adjei A., Xu W. (2013). Genetic sequence variants in vitamin D metabolism pathway genes, serum vitamin D level andoutcome in head and neck cancer patients. Int. J. Cancer.

[45] Woodson K., Ratnasinghe D., Bhat N.K., Stewart C., Tangrea J.A., Hartman T.J., Stolzenberg-Solomon R., Virtamo J., Taylor P.R., Albanes D. (1999). Prevalence of disease-related DNA polymorphisms among participants in a large cancer prevention trial. Eur. J. Cancer Prev..

[46] Ramnath N., Daignault-Newton S., Dy G.K., Muindi J.R., Adjei A., Elingrod V.L., Kalemkerian G.P., Cease K.B., Stella P.J., Brenner D.E. (2013). A phase I/II pharmacokinetic and pharmacogenomic study of calcitriol in combination with cisplatin and docetaxel in advanced non-small-cell lung cancer. Cancer Chemother. Pharmacol..

[47] Kong J., Xu F., Qu J., Wang Y., Gao M., Yu H., Qian B. (2015). Genetic polymorphisms in the vitamin D pathway in relation to lung cancer risk and survival. Oncotarget.

[48] Cheung C.L., Lau K.S., Sham P.C., Tan K.C., Kung A.W. (2013). Genetic variant in vitamin D binding protein is associated with serum 25-hydroxyvitamin D and vitamin D insufficiency in southern Chinese. J. Hum. Genet..

[49] Anic G.M., Thompson R.C., Nabors L.B., Olson J.J., Browning J.E., Madden M.H., Murtagh F.R., Forsyth P.A., Egan K.M. (2012). An exploratory analysis of common genetic variants in the vitamin D pathway including genome-wide associated variants in relation to glioma risk and outcome. Cancer Causes Control.

[50] Jedrzejuk D., Łaczmański Ł., Milewicz A., Kuliczkowska-Płaksej J., Lenarcik-Kabza A., Hirnle L., Zaleska-Dorobisz U., Lwow F. (2015). Classic PCOS phenotype is not associated with deficiency of endogenous vitamin D and *VDR* gene polymorphisms rs731236 (Taq1), rs7975232 (Apa1), rs1544410 (Bsm1), rs10735810 (Fok1): A case-control study of lower Silesian women. Gynecol. Endocrinol..

[51] Kaabachi W., Kaabachi S., Rafrafi A., Amor A.B., Tizaoui K., Haj Sassi F., Hamzaoui K. (2014). Association of vitamin D receptor Fok1 and Apa1 polymorphisms with lung cancer risk in Tunisian population. Mol. Biol. Rep..

[52] Dogan I., Onen H.I., Yurdakul A.S., Konac E., Ozturk C., Varol A., Ekmekci A. (2009). Polymorphisms in the vitamin D receptor gene and risk of lung cancer. Med. Sci. Monit..

[53] Xiong L., Cheng J., Gao J., Wang J., Liu X., Wang L. (2013). Vitamin D receptor genetic variants are associated with chemotherapy response and prognosis in patients with advanced non-small-cell lung cancer. Clin. Lung Cancer.

[54] Fu Y., Li J., Zhang Y. (2014). Polymorphisms in the vitamin D receptor gene and the lung cancer risk. Tumour Biol..

[55] Heist R.S., Zhou W., Wang Z., Liu G., Neuberg D., Su L., Asomaning K., Hollis B.W., Lynch T.J., Wain J.C. (2008). Circulating 25-hydroxyvitamin D, *VDR* polymorphisms, and survival in advanced non-small-cell lung cancer. J. Clin. Oncol..

[56] Ala-Houhala M.J., Vähävihu K., Snellman E., Hasan T., Kautiainen H., Karisola P., Dombrowski Y., Schauber J., Saha H., Reunala T. (2013). A narrow-band ultraviolet B course improves vitamin D balance and alters cutaneous *CYP27A1* and *CYP27B1* mRNA expression levels in haemodialysis patients supplemented with oral vitamin D. Nephron. Clin. Pract..

[57] Jóźwicki W., Brożyna A.A., Siekiera J., Slominski A.T. (2015). Expression of vitamin D receptor (*VDR*) positively correlates with survival of urothelial bladder cancer patients. Int. J. Mol. Sci..

[58] Young M.R., Ihm J., Lozano Y., Wright M.A., Prechel M.M. (1995). Treating tumor-bearing mice with vitamin D_3_ diminishes tumor-induced myelopoiesis and associated immunosuppression, and reduces tumor metastasis and recurrence. Cancer Immunol. Immunother..

[59] Kawar N., Maclaughlan S., Horan T.C., Uzun A., Lange T.S., Kim K.K., Hopson R., Singh A.P., Sidhu P.S., Glass K.A. (2013). PT19c, another nonhypercalcemic vitamin D_2_ derivative, demonstrates antitumor efficacy in epithelial ovarian and endometrial cancer models. Genes Cancer.

[60] Thun M.J., Hannan L.M., Adams-Campbell L.L., Boffetta P., Buring J.E., Feskanich D., Flanders W.D., Jee S.H., Katanoda K., Kolonel L.N. (2008). Lung cancer occurrence in never-smokers: An analysis of 13 cohorts and 22 cancer registry studies. PLoS Med..

[61] Kawanishi S., Hiraku Y., Oikawa S. (2001). Mechanism of guanine-specific DNA damage by oxidative stress and its role in carcinogenesis and aging. Mutat. Res..

[62] Pastoriza Gallego M., Sarasin A. (2003). Transcription-coupled repair of 8-oxoguanine in human cells and its deficiency in some DNA repair diseases. Biochimie.

[63] Lai C.H., Jaakkola J.J., Chuang C.Y., Liou S.H., Lung S.C., Loh C.H., Yu D.S., Strickland P.T. (2013). Exposure to cooking oil fumes and oxidative damages: A longitudinal study in Chinese military cooks. J. Expo. Sci. Environ. Epidemiol..

[64] Fry J.S., Lee P.N., Forey B.A., Coombs K.J. (2013). How rapidly does the excess risk of lung cancer decline following quitting smoking? A quantitative review using the negative exponential model. Regul. Toxicol. Pharmacol..

[65] Maneechay W., Boonpipattanapong T., Kanngurn S., Puttawibul P., Geater S.L., Sangkhathat S. (2015). Single nucleotide polymorphisms in the *GC* gene for vitamin D binding protein in common cancers in Thailand. Asian Pac. J. Cancer Prev..

[66] Anic G.M., Weinstein S.J., Mondul A.M., Männistö S., Albanes D. (2014). Serum vitamin D, vitamin D binding protein, and lung cancer survival. Lung Cancer.

[67] Wang J., Eliassen A.H., Spiegelman D., Willett W.C., Hankinson S.E. (2014). Plasma free 25-hydroxyvitamin D, vitamin D binding protein, and risk of breast cancer in the Nurses’ Health Study II. Cancer Causes Control.

[68] Pande M., Thompson P.A., Do K.A., Sahin A.A., Amos C.I., Frazier M.L., Bondy M.L., Brewster A.M. (2013). Genetic variants in the vitamin D pathway and breast cancer disease-free survival. Carcinogenesis.

[69] Piper M.R., Freedman D.M., Robien K., Kopp W., Rager H., Horst R.L., Stolzenberg-Solomon R.Z. (2015). Vitamin D-binding protein and pancreatic cancer: A nested case-control study. Am. J. Clin. Nutr..

[70] Poynter J.N., Jacobs E.T., Figueiredo J.C., Lee W.H., Conti D.V., Campbell P.T., Levine A.J., Limburg P., Le Marchand L., Cotterchio M. (2010). Genetic variation in the vitamin D receptor (*VDR*) and the vitamin D-binding protein (*GC*) and risk for colorectal cancer: Results from the colon cancer family registry. Cancer Epidemiol. Biomark. Prev..

[71] Flohil S.C., de Vries E., van Meurs J.B., Fang Y., Stricker B.H., Uitterlinden A.G., Nijsten T. (2010). Vitamin D-binding protein polymorphisms are not associated with development of (multiple) basal cell carcinomas. Exp. Dermatol..

[72] Leslie H.S., Mary K.G., Christian W. (2009). TNM Classification of Malignant Tumours.

[73] Wang X., Cui J., Gu J., He H., Li B., Li W. (2015). Plasma 25-hydroxyvitamin D deficiency is associated with the risk of non-small cell lung cancer in a Chinese population. Cancer Biomark..

[74] Schaap M.M., Kunst A.E., Leinsalu M., Regidor E., Ekholm O., Dzurova D., Helmert U., Klumbiene J., Santana P., Mackenbach J.P. (2008). Effect of nationwide tobacco control policies on smoking cessation in high and low educated groups in 18 European countries. Tob. Control.

[75] Wagner D., Hanwell H.E., Vieth R. (2009). An evaluation of automated methods for measurement of serum 25-hydroxyvitamin D. Clin. Biochem..

[76] Carlson C.S., Eberle M.A., Rieder M.J., Yi Q., Kruglyak L., Nickerson D.A. (2004). Selecting a maximally informative set of single-nucleotide polymorphisms for association analyses using linkage disequilibrium. Am. J. Hum. Genet..

[77] Dong J., Hu Z., Wu C., Guo H., Zhou B., Lv J., Lu D., Chen K., Shi Y., Chu M. (2012). Association analyses identify multiple new lung cancer susceptibility loci and their interactions with smoking in the Chinese population. Nat. Genet..

[78] Sugimura H., Tao H., Suzuki M., Mori H., Tsuboi M., Matsuura S., Goto M., Shinmura K., Ozawa T., Tanioka F. (2011). Genetic susceptibility to lung cancer. Front. Biosci..

[79] Reimers L.L., Crew K.D., Bradshaw P.T., Santella R.M., Steck S.E., Sirosh I., Terry M.B., Hershman D.L., Shane E., Cremers S. (2015). Vitamin D-related gene polymorphisms, plasma 25-hydroxyvitamin D, and breast cancer risk. Cancer Causes Control.

[80] Romanos J., van Diemen C.C., Nolte I.M., Trynka G., Zhernakova A., Fu J., Bardella M.T., Barisani D., McManus R., van Heel D.A. (2009). Analysis of HLA and non-HLA alleles can identify individuals at high risk for celiac disease. Gastroenterology.

