# Supplementary material for: Vitamin D-Related Gene Polymorphisms, Plasma 25-Hydroxy-Vitamin D, Cigarette Smoke and Non-Small Cell Lung Cancer (NSCLC) Risk
Source: Int J Mol Sci. 2016 Sep 22;17(10):1597. doi: 10.3390/ijms17101597 (PMC5085630; doi:10.3390/ijms17101597)
Supplement: Supplementary file 1 [file ijms-17-01597-s001.pdf]

Supplementary Materials: Vitamin D-Related Gene Polymorphisms, Plasma 25-Hydroxy-Vitamin D, Cigarette Smoke and Non-Small Cell Lung Cancer (NSCLC) Risk

Xiayu Wu, Jiaoni Cheng and Kaiyun Yang

Table S1. Characteristics of the single-nucleotide polymorphisms (SNPs) and primers, polymerase chain reaction (PCR) conditions used in the study.

SNP [a] Reference	Gene	Chr [b]	Position	Type	W/V [c]	MAF [d]	Primer Sequence	PCR Conditions
rs10735810	*VDR (Fok1)*	12: (46559162)	No block (Exon 4)	Missense	*C/T*	0.41	F:5′-AGCTGGCCCTGGCACTGACTCTGCTCT-3′ R:5′-ATGGAAACACCTTGCTTCTTCTCCCTC-3′	95 °C for 5 min (95 °C for 30 s, 61 °C for 30 s, 72 °C for 30 s) ×40 cycles, 72 °C for 5 min
rs11568820	*VDR (Cdx2)*	12: (46588812)	BlockC2 (Intron 2)	Intron	*T/C*	0.49	F:5′-AGTAAACTAGGTCACAATAAAAACTTATTTCTT-3′ R:5′-AGTAAACTAGGTCACAGTAAAAACTTATTTCTT-3′	95 °C for 5 min (95 °C for 30 s, 56 °C for 30 s, 72 °C for 30 s) ×40 cycles, 72 °C for 5 min
rs1544410	*VDR (Bsm1)*	12: (46526102)	Block B (Intron 2)	Intron	*G/A*	0.11	F:5′-CAGAGCATGGACAGGGAGCAA-3′ R:5′-GCAACTCCTCATGGCTGAGGTCTC-3′	95 °C for 5 min (95 °C for 30 s, 60 °C for 30 s, 72 °C for 30 s) ×40 cycles, 72 °C for 5 min
rs7975232	*VDR (Apa1)*	12: (46525104)	Block B (Intron 8)	Intron	*C/A*	0.44	F:5′-GCTCTCAGCTGGGCCCCTCACTGCTCAAT-3′ R:5′-GCTCTCAGCTGGGCACCTCACTGCTCAAT-3′	95 °C for 5 min (95 °C for 30 s, 70 °C for 30 s, 72 °C for 45 s) ×40 cycles, 72 °C for 5 min
rs731236	*VDR (Taq1)*	12: (46525024)	Block B (Exon 11)	Synonymous	*T/C*	0.13	F:5′-CAGAGCATGGACAGGGAGCAA-3′ R:5′-GCAACTCCTCATGGCTGAGGTCTC-3′	95 °C for 5 min (95 °C for 30 s, 55 °C for 30 s, 72 °C for 45 s) ×40 cycles, 72 °C for 5 min
rs6068816	*CYP24A1*	20: (52214498)	Block B (Exon 6)	Synonymous	*C/T*	0.48	F:5′-ACTTCGTCAGTAACGGACGAGTGA-3′ R:5′-GAGTCGAGGTCATATCGTGAGTGA-3′	95 °C for 5 min (95 °C for 30 s, 60 °C for 30 s, 72 °C for 45 s) ×40 cycles, 72 °C for 5 min
rs2244719	*CYP24A1*	20: (52216265)	Block C (Intron 4)	Intron	*T/C*	0.18	F:5′-ATAGGGTTTTTTTTCCTTCCATTTCATCA-3′ R: 5′-ATAGGGTTTTTTTTTCTTCCATTTCATCA-3′	95 °C for 5 min (95 °C for 30 s, 65 °C for 30 s, 72 °C for 45 s) ×40 cycles, 72 °C for 5 min
rs4809960	*CYP24A1*	20: (52219480)	Block D (Intron 4)	Intron	*T/C*	0.11	F:5′-ACTTCGTCAGTAACGGACGGCAGG-3′ R:5′-GAGTCGAGGTCATATCGTGGCAGGT-3′	95 °C for 5 min (95 °C for 30 s, 60 °C for 30 s, 72 °C for 45 s) ×40 cycles, 72 °C for 5 min

Table S1. *Cont.*

SNP [a] Reference	Gene	Chr [b]	Position	Type	W/V [c]	MAF [d]	Primer Sequence	PCR Conditions
rs2762939	*CYP24A1*	20: (52214658)	Block B (Intron 5)	Intron	*G/C*	0.40	F:5′-CTCTCTCTGCACCGGTCCTGGGAGCAATG-3′ R:5′-CTCTCTCTGCACCGCTCCTGGGAGCAATG-3′	95 °C for 5 min (95°C for 30 s, 52 °C for 30 s, 72 °C for 30 s) ×40 cycles, 72 °C for 5 min
rs2181874	*CYP24A1*	20: (52217885)	No Block (Intron 4)	Intron	*G/A*	0.15	F:5′-GTAACCGGGAGCTGCGGGAATGTATTCAG-3′ R:5′-GTAACCGGGAGCTGTGGGAATGTATTCAG-3′	95 °C for 5 min (95 °C for 30 s, 60 °C for 30 s, 72 °C for 45 s) ×40 cycles, 72 °C for 5 min
rs2296241	*CYP24A1*	20: (52219626)	Block D (Exon 4)	Synonymous	*G/A*	0.49	F:5′-TTGAAGGTCTTGGCCGATTTTATGGGCAG-3′ R:5′-TTGAAGGTCTTGGCTGATTTTATGGGCAG-3′	95 °C for 5 min (95 °C for 30 s, 65 °C for 30 s, 72 °C for 30 s) ×40 cycles, 72 °C for 5 min
rs10877012	*CYP27B1*	12: (56448352)	No block	downstream	*G/T*	0.41	F:5′-CTGGCACAGAGCCTCATAG-3′ R:5′-CGTATGCCTGTAGTGCCTT-3′	95 °C for 5 min (95 °C for 30 s, 56 °C for 30 s, 72 °C for 30 s) ×35 cycles, 72 °C for 5 min
rs3782130	*CYP27B1*	12: (56448165)	Block A	downstream	*C/G*	0.39	F:5′-GGCCCTCTCTCTGTTGCCTAG-3′ R:5′-CAACACTTTGGGAGGCTAAGG-3′	95 °C for 5 min (95 °C for 30 s, 58 °C for 30 s, 72 °C for 30 s) ×35 cycles, 72 °C for 5 min
rs7041	*GC*	4: (72837198)	Block A (Exon 11)	Missense	*T/G*	0.36	F:5′-AAATAATGAGCAAATGAAAGAAGAC-3′ R:5′-CAATAACAGCAAAGAAATGAGTAGA-3′	95 °C for 3min (95 °C for 30 s, 64 °C for 30 s, 72 °C for 30 s) ×35 cycles, 72 °C for7 min
rs4588	*GC*	4: (72837187)	Block A (Exon 11)	Missense	*C/A*	0.28	F:5′-AAATAATGAGCAAATGAAAGAAGAC-3′ R:5′-CAATAACAGCAAAGAAATGAGTAG-3′	95 °C for 3min (95 °C for 30 s, 64 °C for 30 s, 72 °C for 30 s) ×35 cycles, 72 °C for 7 min

[a] http://hapmap.org; [b] Chr (Chromosome); [c] W/V (Wild/Variation); [d] MAF (Minor Allele Frequency) among controls.

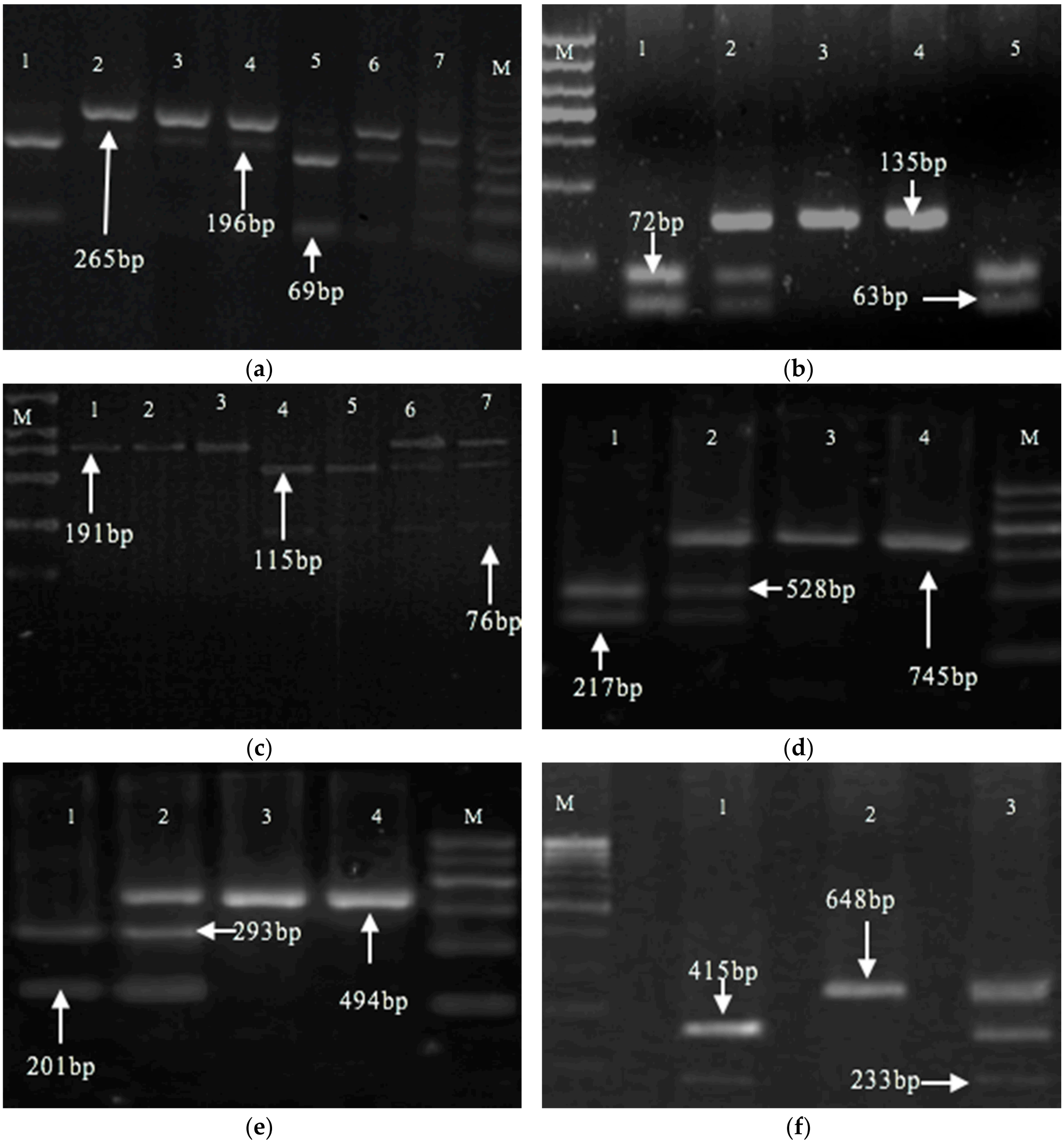

Figure S1. *Cont.*

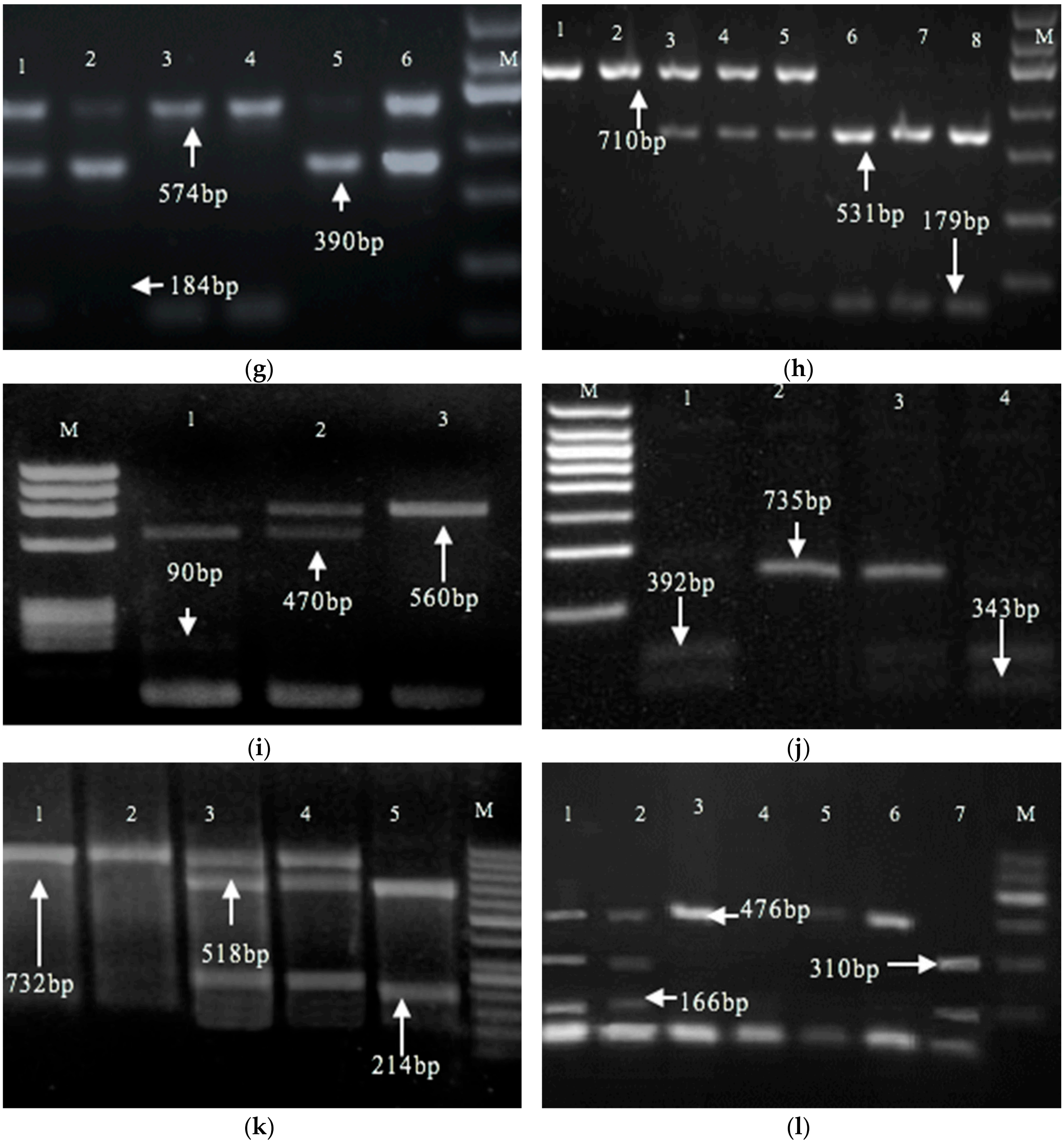

Figure S1. *Cont.*

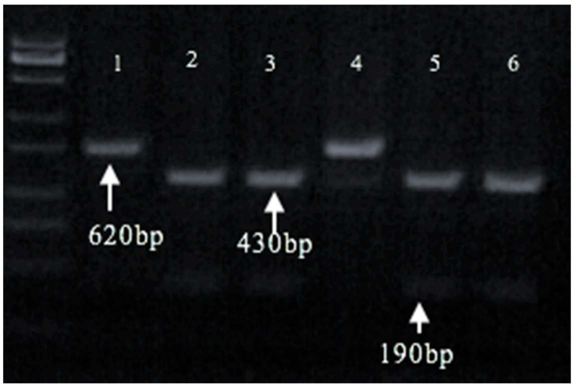

(**m**)

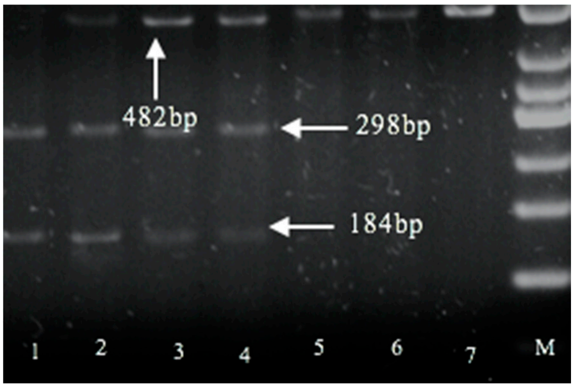

(**n**)

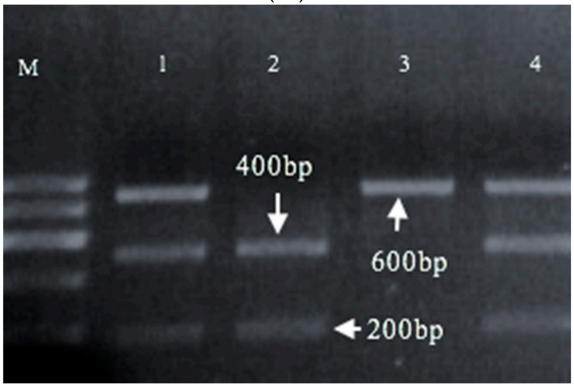

(**o**)

Figure S1. Genotyping of the Vitamin D-related gene polymorphisms Polymorphism by PCR-RFLP. (**a**) Genotyping of the *VDR* rs10735810 (*Fok1*) Polymorphism by PCR-RFLP. Lanes 2 and 3 CC (265 bp); Lanes 1 and 5 TT (196 bp and 69 bp); Lanes 4, 6 and 7 CT (265 bp, 196 bp and 69 bp); (**b**) Genotyping of the *VDR* rs11568820 (*Cdx2*) Polymorphism by PCR-RFLP. Lanes 3 and 4 TT (135 bp); Lanes 1 and 5 CC (72 bp and 63 bp); Lane 2 TC (135 bp, 72 bp and 63 bp); (**c**) Genotyping of the *VDR* rs1544410 (*Bsm1*) Polymorphism by PCR-RFLP. Lanes 1, 2 and 3 GG (191 bp); Lanes 4 and 5 AA (115 and 76 bp); Lanes 6 and 7 GA (76, 115 and 191 bp); (**d**) Genotyping of the *VDR* rs7975232 (*Apa1*) Polymorphism by PCR-RFLP. Lanes 3 and 4 CC (745 bp); Lane 1 AA (528 and 217 bp); Lane 2 CA (745, 528 and 217 bp); (**e**) Genotyping of the *VDR* rs731236 (*Taq1*) Polymorphism by PCR-RFLP. Lanes 3 and 4 TT (494bp); Lane 1 CC (201 and 293 bp); Lane 2 TC (201, 293 and 494 bp); (**f**) Genotyping of the *CYP24A1* rs6068816 Polymorphism by PCR-RFLP. Lane 2 CC (648 bp); Lane 1 TT (415 and 233 bp); Lane 3 CT (648, 415 and 233 bp); (**g**) Genotyping of the *CYP24A1* rs2244719 Polymorphism by PCR-RFLP. Lanes 3 and 4 TT (574 bp); Lane 5 CC (390 and 184 bp); Lanes 1, 2 and 6 TC (574, 390 and 184 bp); (**h**) Genotyping of the *CYP24A1* rs4809960 Polymorphism by PCR-RFLP. Lanes 1 and 2 TT (710 bp); Lanes 6, 7 and 8 CC (531 and 179 bp); Lanes 9; 3, 4 and 5 TC (710, 531 and 179 bp); (**i**) Genotyping of the *CYP24A1* rs2762939 Polymorphism by PCR-RFLP. Lane 3 GG (560 bp); Lane 1 CC (470 and 90 bp); Lane 2 GC (560, 470 and 90 bp); (**j**) Genotyping of the *CYP24A1* rs2181874 Polymorphism by PCR-RFLP. Lane 2 GG (735 bp); Lanes 1 and 4 AA (392 and 343 bp); Lane 3 GA (735, 392 and 343 bp); (**k**) Genotyping of the *CYP24A1* rs2296241 Polymorphism by PCR-RFLP. Lanes 1 and 2 *GG* (732 bp); Lane 5 AA (518 and 214 bp); Lanes 3 and 4 GA (574, 390 and 184 bp); (**l**) Genotyping of the *CYP27B1* rs10877012 Polymorphism by PCR-RFLP. Lanes 3, 4, 5, 6 GG (476 bp); Lanes 7 TT (310 and 166 bp); Lanes 1, 2 GT (476, 310 and 166 bp); (**m**) Genotyping of the *CYP27B1* rs3782130 Polymorphism by PCR-RFLP. Lane 1 CC (620 bp); Lanes 2, 3, 5 and 6 GG (430 and 190 bp); Lane 4 CG (620, 430 and 190 bp); (**n**) Genotyping of the GC rs7041 Polymorphism by PCR-RFLP. Lanes 5, 6 and 7 TT (482 bp); Lanes 1 and 2 GG (298 and 184 bp); Lanes 3 and 4 TG (482, 298 and 184 bp); (**o**) Genotyping of the GC rs4588 Polymorphism by PCR-RFLP. Lane 3 CC (600 bp), Lane 2 AA (400 and 200 bp), Lanes 1 and 4 CA (600, 400 and 200 bp).
